# Dissecting the role of strigolactone perception in barley tolerance to cadmium or zinc stress based on the receptor mutant analysis

**DOI:** 10.1186/s12870-025-07683-4

**Published:** 2025-11-21

**Authors:** Weronika Buchcik, Krzysztof Sitko, Marek Marzec

**Affiliations:** Institute of Biology, Biotechnology and Environmental Protection, Faculty of Natural Sciences, University of Silesia in Katowice, Jagiellonska 28, 40‑032, Katowice, Poland

**Keywords:** Barley, Cadm, DWARF14, Receptor, Strigolactones, Transcriptome, Zinc

## Abstract

Strigolactones (SLs) are plant hormones involved in development and environmental responses, but their role in heavy metal stress tolerance has not been fully characterized. We examined how SL perception affects barley (*Hordeum vulgare*) responses to cadmium (Cd) or zinc (Zn) toxicity by comparing wild-type (WT) plants and the SL-insensitive *hvd14.d* mutant. Phenotypic, ionomic, and transcriptomic analyses revealed that hvd14.d is more sensitive to both metals, exhibiting greater growth inhibition, increased Cd accumulation, altered Zn partitioning, and reduced photosynthetic performance. Elevated oxidative stress markers and weaker antioxidant responses further reflected an impaired redox balance in the mutant. Transcriptomic profiling revealed that SL signaling influences genes controlling detoxification, metal transport, and reactive oxygen species (ROS) homeostasis. Genes associated with ABA signaling were less responsive in *hvd14.d*, suggesting that SL perception contributes to the coordination of ABA-related pathways during metal stress. Transcription factor (TF) analyses identified distinct but overlapping groups of SL-dependent TFs under Cd and Zn exposure, including shared regulators. To date, most studies have focused on exogenous application of synthetic SLs; this work is the first to examine the role of endogenous SL perception in barley responses to heavy metal stress. Our findings demonstrate that SL perception influences both transcriptional programs and physiological processes essential for metal tolerance and redox regulation.

**Supplementary Information:**

The online version contains supplementary material available at 10.1186/s12870-025-07683-4.

## Introduction

Strigolactones (SLs) are a class of carotenoid-derived phytohormones, first identified for their rhizospheric role as germination stimulants of parasitic plants [1] but now recognized as key regulators of plant development and stress adaptation. In 2008, the involvement of SLs in shaping shoot architecture in both monocots and dicots was proposed, and mutations in SL biosynthesis and signaling genes were shown to result in the production of an increased number of shoot branches [2, 3]. Next, SLs play roles in multiple aspects of plant development. They regulate root architecture by inhibiting lateral root formation while promoting primary root elongation and root hair development [4, 5]. SLs are also involved in the regulation of leaf senescence, with SL mutants displaying delayed aging phenotypes [6], as well as influencing internode elongation [7] and photomorphogenesis [8].

Recent studies show that SLs modulate plant responses to various abiotic and biotic stresses. Under nutrient-deficient conditions, particularly phosphate and nitrogen starvation, SL biosynthesis and exudation are significantly upregulated, leading to adaptive modifications in the root system architecture to enhance nutrient uptake. SLs also mediate long-distance signaling under such conditions, coordinating shoot and root responses to maintain growth under nutrient limitation [9]. Beyond nutrient stress, SLs have been shown to play a role in plant responses to drought and salinity. In Arabidopsis (*Arabidopsis thaliana*) [10–12], tomato (*Solanum lycopersicum*) [13, 14] and barley (*Hordeum vulgare*) [15, 16] SL-deficient or SL-insensitive mutants exhibit increased sensitivity to drought due to altered stomatal behavior, reduced water-use efficiency, and less sensitivity to ABA. Moreover, SLs contribute to the regulation of antioxidant defense mechanisms, contributing to ROS detoxification under stress [16–18].

 In silico analyses suggest that SLs may also be involved in plant responses to flooding and heavy metal stress [19, 20]. In recent years, the first experimental reports have emerged confirming that SLs may play a pivotal role in enhancing plant tolerance to heavy metals. Cadmium (Cd) and zinc (Zn) are non-essential and essential heavy metals, respectively, that significantly impact plant physiology depending on their concentration and bioavailability. While Zn is an indispensable micronutrient involved in numerous enzymatic and structural functions, excessive Zn accumulation disrupts metabolic processes and induces oxidative stress [21]. In contrast, Cd is a toxic metal that is not required for plant development and is readily absorbed via transporters for essential divalent cations such as iron (Fe²⁺) [22]. Cd exposure impairs root elongation, disrupts the photosynthetic machinery, promotes lipid peroxidation, and leads to an overproduction of ROS, ultimately affecting plant growth and yield [23].

Several recent studies have highlighted the functional role of SLs in modulating plant tolerance to Cd stress. Application of GR24, a synthetic SL analog, has been shown to reduce Cd toxicity in various mono- and dicot species. Treatment with GR24 (synthetic SL analog) mitigated Cd-induced phytotoxicity in *Artemisia annua* by reducing ROS accumulation, enhancing antioxidant enzyme activities, maintaining chloroplast ultrastructure, and sustaining the biosynthesis of secondary metabolites such as artemisinin [24]. In wheat (*Triticum aestivum*), GR24 reduced Cd uptake, promoted nitric oxide (NO) signaling, and activated key antioxidant genes, leading to improved photosynthetic efficiency and reduced lipid peroxidation [25]. Similar responses were documented in barley (*Hordeum vulgare*), where synthetic SLs promoted the accumulation of photosynthetic pigments, regulated antioxidant metabolism, and modulated Cd allocation between roots and shoots [17]. Furthermore, soybean (*Glycine max*) plants subjected to Cd stress showed lower metal translocation, enhanced glyoxalase defense system, and increased nodulation and seed yield following GR24 application [26]. Combinatorial strategies using SLs and biochar demonstrated synergistic effects in radish (*Raphanus sativus*), where Cd uptake was significantly reduced, ROS and lipid peroxidation levels decreased, and growth parameters improved [27]. These findings suggest that SLs modulate heavy metal stress responses at different levels, including metal uptake, ROS scavenging, and maintenance of metabolic homeostasis.

Despite these insights, critical knowledge gaps remain. Most studies have focused on the application of synthetic SLs, and the role of endogenous SL perception in heavy metal stress responses remains unknown. Notably, to date, no research has evaluated how genetic impairment of SL perception through mutations in the SL receptor (DWARF14, D14) affects Cd stress tolerance. Moreover, no data are available for other heavy metals, such as Zn, for which plants may have developed distinct defense mechanisms.

Barley is an appropriate model for these investigations. It is both a globally important cereal crop [28] and a species sensitive to Cd and Zn excess [29]. Also, for barley, the SL receptor DWARF14 (HvD14) has been identified and functionally characterized, enabling genetic dissection of SL signaling. A well-characterized *hvd14.d* loss-of-function mutant (carrying a point mutation in HvD14) exhibits the classic SL-insensitive phenotypes, it is semi-dwarf with increased tillering and an altered root system as well as does not respond to exogenous SL analog treatment [30]. This mutant has become a key tool for elucidating SL functions in barley, revealing roles that extend beyond development. Under drought stress, *hvd14.d* plants exhibit heightened sensitivity associated with impaired abscisic acid (ABA)-dependent stomatal regulation, indicating that SL perception via HvD14 is essential for a normal drought response [15]. Consistently, multi-omics analyses have demonstrated that intact SL signaling confers a distinct advantage during water deficit, coordinating extensive changes in gene expression and metabolism that underlie improved drought tolerance [16]. Even under non-stress conditions, loss of SL perception causes broad transcriptomic reprogramming in both shoots and roots [31] and perturbs hormonal homeostasis [32], indicating that SL interacts with multiple hormonal pathways to maintain developmental and physiological balance. Moreover, SL signaling influences key agronomic traits: the *hvd14.d* mutant produces an excess number of tillers but with reduced grain size, a trade-off suggesting that SLs coordinate tillering and grain-filling processes [33]. Together, these findings highlight the crucial role of HvD14-mediated SL perception in determining growth, stress resilience, and yield-related traits in barley. Therefore, genetic dissection using barley mutant impaired in SL perception provides a tool to dissect the regulatory role of SL signaling under heavy metal stress. In this study, we investigated the role of SL perception in barley responses to Cd or Zn toxicity using wild-type plants and SL receptor mutant via physiological and transcriptomic studies. Our findings will provide novel insights into the endogenous SL contributions to plant tolerance mechanisms against heavy metals.

## Materials and methods

### Plant material and growth conditions

The plant material used in this study consisted of barley (*Hordeum vulgare* L.) SL-insensitive mutant *hvd14.d* and its wild-type parent cultivar Sebastian. The *hvd14.d* mutant, impaired in SL signaling [30], was isolated from an M_2_ population of Sebastian generated by chemical mutagenesis using the Targeted Induced Local Lesions in Genomes (TILLING) approach [34]. The mutant carries a G725A point mutation in the HvDWARF14 gene (NCBI accession number: KP069479), which encodes the SL receptor.

Seeds of both genotypes were sown on filter paper moistened with deionized water and germinated in the dark for 4 days at 21 °C in full humidity. Then, the seedlings were transferred to a greenhouse at 21 °C and illuminated with HPS lamps at 150 PAR in a 16-hour day/8-hour night system. Four-day-old seedlings were placed in 2000 ml hydroponic containers, six seedlings per container. The plants were grown on modified Hoagland medium consisting of: 16 mM N, 6 mM K, 4 mM Ca, 2 mM Mg, 2 mM S, 1 mM P, 50 µM Fe, 25 µM B, 2 µM Mn, 2 µM Zn, 2 µM Cl, 0.5 µM Cu, and 0.5 µM Mo per liter. The pH of the medium was set at 5.8 + 0.1. The medium was changed twice a week. After ten days of growth in control conditions, the containers were divided into variants: control, treated with Cd at a concentration of 5 µM, or treated with Zn at a concentration of 50 µM. The treatment lasted another ten days. On the last day of the experiment, selected physiological parameters of plants were measured, and biomass was collected for further analyses.

### Physiological parameters’ measurements

A fully developed leaf (the third, counted from the seed’s bottom) was selected for measurements of selected growth and physiological parameters. For each repetition, measurements were performed within two days. Measurements were made on the same leaf for each individual in hydroponic culture.

The pigment content meter (Dualex Scientific+, Force-A, Orsay, France) was used to measure the content of chlorophyll, flavonols, and anthocyanins in leaves. The measurements were taken after the device was automatically calibrated by placing a leaf blade between the measuring heads. Chlorophyll *a* fluorescence measurements were conducted for the same seedlings using the Plant Efficiency Analyser (PocketPEA fluorimeter; Hansatech Ltd., Pentney, UK). Before being measured, each selected leaf was adapted in the dark for 30 min using leaf clips. After adaptation, a saturating light pulse of 3500 mmol quanta m^− 2^ s^− 1^ was applied for 1 s, which closed all of the reaction centres, and the fluorescence parameters were measured. Leaf gas exchange parameters were measured using LCpro-SD infra-red gas analyser (ADC Bioscientific Ltd., Hoddesdon, GB). External light at 1000 PAR was given to the chamber, and parameters were measured after at least 1.5 min of incubation in the measuring chamber and stabilization of the reading.

The plant shoot and root material were acid-digested in a microwave-assisted wet digestion system (ETHOS 1, Milestone, Sorisole, Italy) according to the manufacturer’s procedure (concentrated HNO_3_ and H_2_O_2_, 4:1 v/v). The concentration of metals was analysed in the extracts (soil, CaCl_2_) and digests (plant, soil) using flame atomic absorption spectrophotometry (iCE 3500 FAAS, Thermo Fisher Scientific, Waltham, MA, USA). A reference plant (Oriental Basma Tobacco Leaves (INCT-OBTL-5), Institute of Nuclear Chemistry and Technology, Warszawa, Poland) and soil material (NCS DC 77302, China 502 National Analysis Center for Iron and Steel, Beijing, China) were used to determine the quality assurance of the analytical data.

### RNA isolation, library preparation, and transcriptome analysis

Root and shoot samples were collected from control and stressed plants. Each biological replicate consisted of pooled tissue from four plants, and four independent replicates were obtained per genotype. Total RNA was extracted using the mirVana miRNA Isolation Kit (ThermoFisher Scientific), and its quality was verified prior to library construction.

Polyadenylated transcripts were isolated from total RNA using oligo(dT) magnetic beads and fragmented. Strand-specific libraries were generated through reverse transcription, second-strand synthesis incorporating dUTP, end repair, adapter ligation, USER enzyme digestion, and PCR amplification. Library quality was confirmed using Qubit, qPCR, and Bioanalyzer analyses. Qualified libraries were pooled and sequenced on the Illumina platform (Novogene).

Raw reads were processed to remove adapters, poly-N sequences, and low-quality reads. Clean reads were assessed for base quality (Q20, Q30) and GC content. High-quality reads were then aligned to the barley reference genome (Ensembl Plants, TAIR v10) using HISAT2 (v2.0.5) with default parameters and gene model annotations.

Read counts per gene were quantified using featureCounts (v1.5.0-p3). Gene expression levels were calculated as FPKM values, normalized for gene length and sequencing depth. Differential expression analysis was performed using DESeq2 (v1.20.0), based on raw counts. Genes with an adjusted P-value (FDR) ≤ 0.05 were classified as significantly differentially expressed.

### PCA analysis of RNA-seq expression data

RNA-seq data were obtained from *Hordeum vulgare* wild-type (WT) and *hvd14.d* mutant plants exposed to Cd or Zn. Transcript abundance was quantified as fragments per kilobase of transcript per million mapped reads (FPKM). Four biological replicates were collected for each combination of genotype (WT, *hvd14.d*) and tissue (root, shoot). FPKM values were log-transformed and filtered to remove genes with zero expression across all samples. The data were then standardized using z-score normalization. PCA was performed on the scaled expression matrix using the scikit-learn Python package, and the resulting principal components were visualized using seaborn and matplotlib.

### TF prediction and identification of TF binding sites

Amino acid sequences of DEGs were retrieved from the *Hordeum vulgare* genes (IBSC v2) dataset using the BioMart tool (https://plants.ensembl.org/biomart). These sequences were subsequently used as queries in the TF Prediction tool available at PlantTFDB (http://planttfdb.gao-lab.org/prediction.php).

Promoter regions (1500 bp upstream of the start codon) for DEGs and DAPs were also extracted from the same dataset via BioMart. The extracted promoter sequences served as input for the Binding Site Prediction tool in PlantTFDB (http://plantregmap.gao-lab.org/binding_site_prediction.php), with *Hordeum vulgare* selected as the reference and a significance threshold of *p* ≤ 1e − 4 applied.

## Results

### Effect of cd or Zn on barley development

Under control conditions, the *hvd14.d* mutant displayed a markedly altered growth phenotype compared to the wild-type (WT) cultivar Sebastian. Mutant plants were significantly shorter, produced a greater number of tillers, and exhibited reduced root length (Table 1, Fig. S1), confirming the regulatory role of SL signaling in shoot and root development as proposed previously [30, 31]. Exposure to Cd (5 µM) or Zn (50 µM) affected the barley growth and development in a metal- and genotype-dependent manner. The *hvd14.d* mutant consistently exhibited lower shoot growth than WT, regardless of treatment. Cd did not exert a significant inhibitory effect on shoot elongation in either genotype. In contrast, Zn application induced a hormetic response in WT plants, leading to a modest but significant increase in shoot length in shoot length, while no such effect was observed in the mutant (Table 1, Fig. S1). Shoot fresh weight was not significantly affected by either metal in WT plants. However, in the *hvd14.d* mutant, a pronounced reduction in shoot biomass was observed under both Cd and Zn treatment. A similar trend was observed for the number of tillers: metal exposure did not significantly impact WT, whereas the mutant showed a marked decrease in tiller number relative to control conditions (Table 1, Fig. S1).

**Table 1:** Growth and ionomics of barley WT and *hvd14.d* mutant treated with Cd or Zn compared to the control

genotype	WT			*hvd14.d*		
*treatment*	control	+ Zn	+ Cd	control	+ Zn	+ Cd
*Growth parametrs*						
Shoot lenght (cm)	33.8 ± 0.7b	38.6 ± 0.6a	33.7 ± 0.4b	31.7 ± 0.7c	31.3 ± 0.6c	30.3 ± 0.5c
Root lenght (cm)	31.7 ± 0.7a	25.4 ± 0.8bc	22.2 ± 1.3 cd	23.2 ± 1.5c	19.7 ± 1.1d	26.2 ± 0.9b
Number of tillers	3.9 ± 0.2b	4.0 ± 0.1b	4.0 ± 0.1b	4.6 ± 0.1a	4.0 ± 0.1b	4.2 ± 0.1ab
Shoots FW (g)	3.2 ± 0.1a	3.0 ± 0.1a	3.1 ± 0.1a	2.0 ± 0.1b	1.6 ± 0.1c	1.5 ± 0.1c
Roots FW (g)	1.1 ± 0.1a	0.9 ± 0.0b	1.0 ± 0.1ab	0.7 ± 0.0c	0.5 ± 0.0d	0.6 ± 0.0 cd
Leaf thickness (mm)	0.19 ± 0.02b	0.18 ± 0.01b	0.27 ± 0.03a	0.21 ± 0.01b	0.13 ± 0.01c	0.16 ± 0.01bc
*Element concentration in leaves (µg g*^*− 1*^ *DW)*						
Ca	2490 ± 530ab	2460 ± 480ab	1700 ± 350b	3920 ± 450a	2990 ± 600ab	3380 ± 560a
Cd	BDL	BDL	11.9 ± 0.9b	BDL	BDL	15.0 ± 0.8a
Fe	93 ± 89a	90 ± 12ab	67 ± 2b	75 ± 7ab	85 ± 9ab	71 ± 4ab
K	52,900 ± 1700a	51,000 ± 3100ab	48,100 ± 2100ab	48,400 ± 2200ab	47,200 ± 2100ab	45,000 ± 1400b
Mg	920 ± 30b	770 ± 90b	770 ± 40b	1230 ± 60a	1320 ± 130a	1180 ± 70a
Mn	35 ± 3ab	35 ± 5ab	23 ± 5b	41 ± 4a	31 ± 3ab	23 ± 3b
Zn	10 ± 1c	81 ± 3a	9 ± 1c	3 ± 1d	74 ± 1b	2 ± 0d
*Element concentration in roots (µg g*^*− 1*^ *DW)*						
Ca	820 ± 110c	1030 ± 90bc	1490 ± 40a	1330 ± 120ab	1400 ± 210ab	1720 ± 90a
Cd	BDL	BDL	260.3 ± 21.3b	BDL	BDL	507.0 ± 58.9a
Fe	10,360 ± 950a	7550 ± 1250b	9280 ± 380ab	8680 ± 300ab	9660 ± 680ab	11,830 ± 1250a
K	46,100 ± 5400ab	42,700 ± 3500ab	38,200 ± 2100b	45,100 ± 2300ab	47,300 ± 4400ab	52,900 ± 4100a
Mg	700 ± 50abc	660 ± 70abc	850 ± 100a	560 ± 30c	730 ± 40ab	750 ± 30ab
Mn	311 ± 13a	111 ± 5b	72 ± 10b	256 ± 28a	126 ± 10b	70 ± 23b
Zn	28 ± 6c	530 ± 51b	22 ± 3c	50 ± 8c	1034 ± 84a	41 ± 16c

Data are means ± SE (*n* = 18 or 5, for growth or ionomics, respectively)

Lowercase letters describe statistical significance in a row (one-way Anova, LSD test, *P* < 0.05)

*Abbreviations*: *Fw * fresh weight, *DW* dry, *BDL *below detection limit

Root development was more sensitive to metal stress. In WT plants, both Cd and Zn significantly reduced root length and biomass. In contrast, the *hvd14.d* mutant showed root growth inhibition only under Zn treatment, suggesting differential sensitivity and signaling in response to different metals (Table 1, Fig. S1).

### Differential accumulation of metal ions in response to cd or Zn treatment

Ionomic profiling revealed significant genotype-dependent differences in the accumulation of heavy metals and essential nutrients. The *hvd14.d* mutant accumulated significantly more Cd in shoots and nearly twice as much in roots compared to the WT (Table 1). In contrast, shoot Zn concentration was significantly lower in the mutant than in WT plants, whereas root Zn content was approximately twofold higher in *hvd14.d* than in WT, both under control and Zn treatment conditions (Table 1). Cd exposure led to a significant reduction in calcium (Ca) and iron (Fe) levels in the leaves, as well as a decrease in manganese (Mn) content in the roots of WT. Similarly, both genotypes exhibited reduced Mn accumulation in roots under Cd or Zn stress. In *hvd14.d* Cd treatment also caused a notable reduction in Mn concentration, indicating disrupted micronutrient homeostasis (Table 1).

### Impact of cd or Zn on leaf pigmentation and photosynthetic performance

The effects of Cd or Zn on photosynthetic pigments and gas exchange parameters varied between the genotypes. In WT plants, neither metal significantly altered chlorophyll content. However, Cd exposure induced a significant increase in anthocyanin levels and a decrease in flavonol content in WT leaves (Table 2). In contrast, the *hvd14.d* mutant was more sensitive to metal-induced pigment changes. Both Cd or Zn treatments significantly reduced chlorophyll content in mutant leaves relative to the control. Moreover, both metals increased anthocyanin accumulation, while only Cd led to a significant reduction in flavonol content (Table 2).

**Table 2:** Changes in leaf pigments and gas exchange parameters in barley WT and *hvd14.d* mutant treated with cd or Zn compared to the control

cv.	WT			*hvd14.d*		
*treatment*	control	+ Zn	+ Cd	control	+ Zn	+ Cd
*Content of leaf pigments (r.u.)*						
Chlorophyll	43.6 ± 0.5ab	42.3 ± 0.7bc	43.8 ± 0.5a	43.9 ± 0.5a	39.0 ± 0.6d	41.7 ± 0.5c
Flavonol	0.21 ± 0.01ab	0.23 ± 0.01a	0.18 ± 0.01 cd	0.20 ± 0.00bc	0.18 ± 0.01 cd	0.17 ± 0.01d
Anthocyanins	0.106 ± 0.001d	0.106 ± 0.002 cd	0.111 ± 0.001bc	0.102 ± 0.002d	0.119 ± 0.003a	0.115 ± 0.001ab
*Gas exchange parameters*						
A (µmol CO_2_ m^− 2^ s^− 1^)	14.4 ± 0.3a	10.6 ± 0.2c	9.3 ± 0.3e	11.7 ± 0.2b	10.0 ± 0.1 cd	9.4 ± 0.2de
E (mmol H_2_O m^− 2^ s^− 1^)	1.65 ± 0.03a	1.12 ± 0.04c	0.91 ± 0.03d	1.23 ± 0.02b	1.21 ± 0.02b	1.11 ± 0.04c
gs (mmol H_2_O m^− 2^ s^− 1^)	124 ± 2a	80 ± 3c	64 ± 3d	91 ± 2b	85 ± 2bc	78 ± 3c

Data are means ± SE (*n* = 9 or 21, for fluorescence or gas exchange, respectively)

*Abbreviations*: *A* photosynthetic rate, *E* transpiration rate, *gs* stomatal conductance

Lowercase letters describe statistical significance in a row (one-way Anova, LSD test, *P* < 0.05)

Gas exchange measurements indicated that both Cd or Zn strongly inhibited CO₂ assimilation in both genotypes. Cd treatment reduced the photosynthetic rate by 35% in WT and by 20% in *hvd14.d* plants compared to the respective controls. Additionally, both metals significantly suppressed stomatal conductance and transpiration in WT plants. In *hvd14.d*, these processes were significantly inhibited only under Cd treatment, indicating a differential physiological response to Zn (Table 2).

### Effect of Zn or cd on the light phase of photosynthesis

**Fig. 1:**
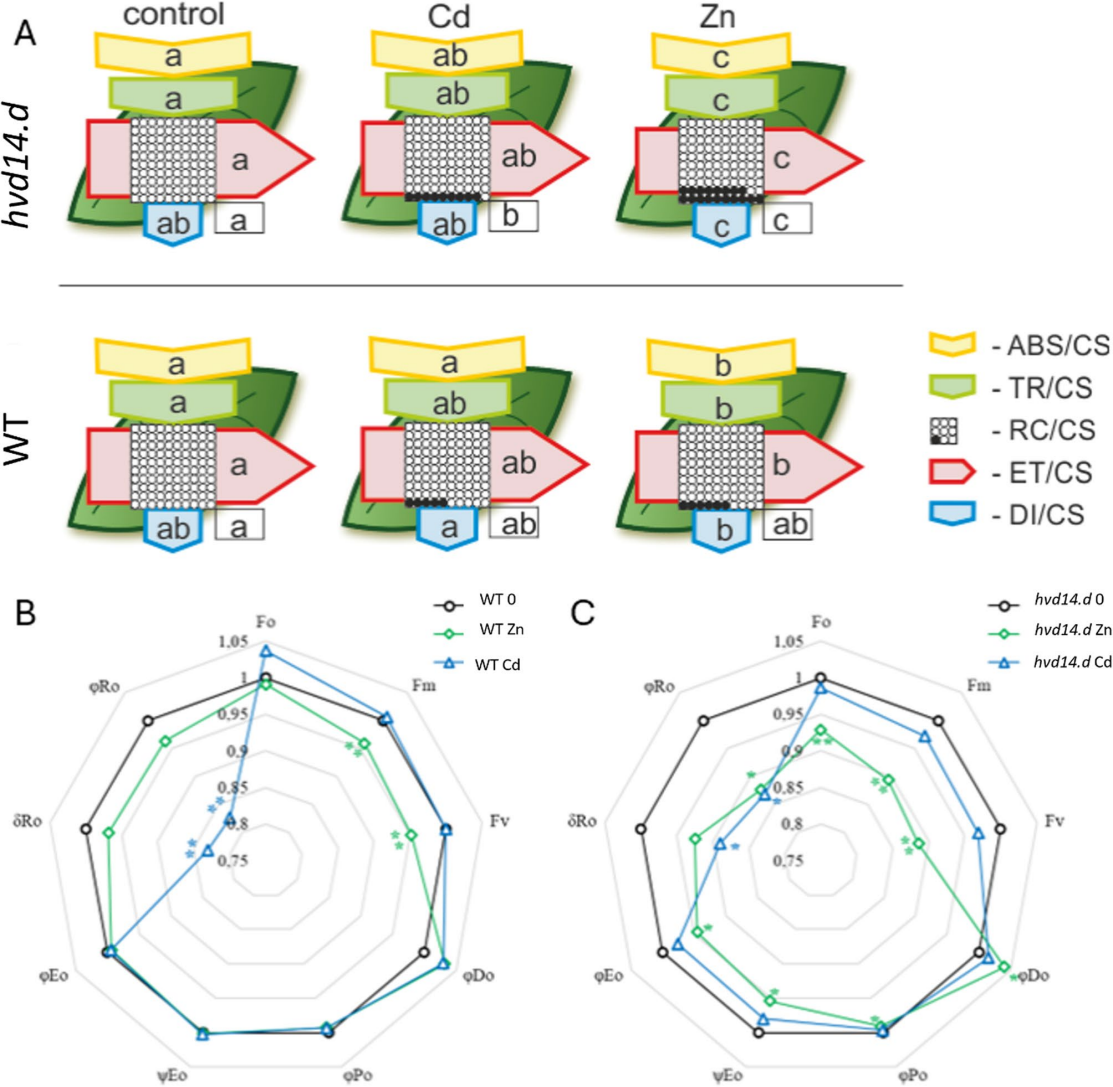
Impact of Zn or Cd treatment on the photosynthetic performance of barley leaves. The leaf pipeline model of energy fluxes through PSII (**A**) of excited cross sections (CS) in barley leaves or changes in selected parameters of chlorophyll fluorescence in WT (**B**) or *hvd14.d* mutant (**C**) treated with Zn or Cd, compared to the control. Each relative value of the measured parameters is the mean (*n* = 9), and each arrow’s width corresponds to the flux’s intensity. Yellow arrow-ABS/CS, absorption flux per CS approximated; green arrow-TR/CS, trapped energy flux per CS; red arrow-ET/CS, electron transport flux per CS; blue arrow-DI/CS, dissipated energy flux per CS; circles inscribed in squares-RC/CS, % of active/inactive reaction centers. White circles inscribed in squares represent reduced Q_A_ reaction centers (active), black (or orange) circles represent non-reduced Q_A_ reaction centers (inactive), 100% of the active reaction centers responded with the highest mean value observed in the control conditions. Means followed by the same letter for each parameter are not significantly different from each other using the Fisher LSD test (*P* < 0.05). Letters are inscribed into arrows, except for RC/CS, where they are placed in a box in the bottom right corner of the square with circles

The impact of Cd or Zn on the light phase of photosynthesis revealed genotype-specific disturbances in photosynthetic energy flow. In WT plants, Cd accumulation did not significantly alter parameters associated with energy fluxes through photosystem II (PSII) (Fig. 1A). In contrast, Zn treatment led to a decrease in both the energy absorbed and trapped by antenna complexes, and significantly reduced electron transport efficiency through PSII, while not affecting the proportion of active reaction centers. In the *hvd14.d* mutant, Cd exposure resulted in a significant decrease in the percentage of active PSII reaction centers relative to control conditions. Zn treatment in the mutant had a more pronounced effect, significantly reducing all major parameters describing energy fluxes per excited cross-section, indicating severe photoinhibition (Fig. 1A).

Chlorophyll fluorescence analyses further confirmed these findings. In WT, Zn exposure caused significant reductions in both maximum (Fm) and variable fluorescence (Fv), reflecting compromised PSII efficiency (Fig. 1B). Cd stress, on the other hand, decreased parameters related to PSI electron transport efficiency and acceptor-side activity (φRo and δRo). Similarly, in the *hvd14.d* mutant, Zn treatment significantly decreased PSII quantum efficiency (φPo), electron transport efficiency (φEo), and the probability of electron transfer beyond QA (ΨEo), consistent with substantial PSII damage (Fig. 1C). Cd treatment in the mutant led to declines in δRo and φRo, mirroring the response observed in the WT.

### Barley transcriptome response to cd treatment

**Fig. 2:**
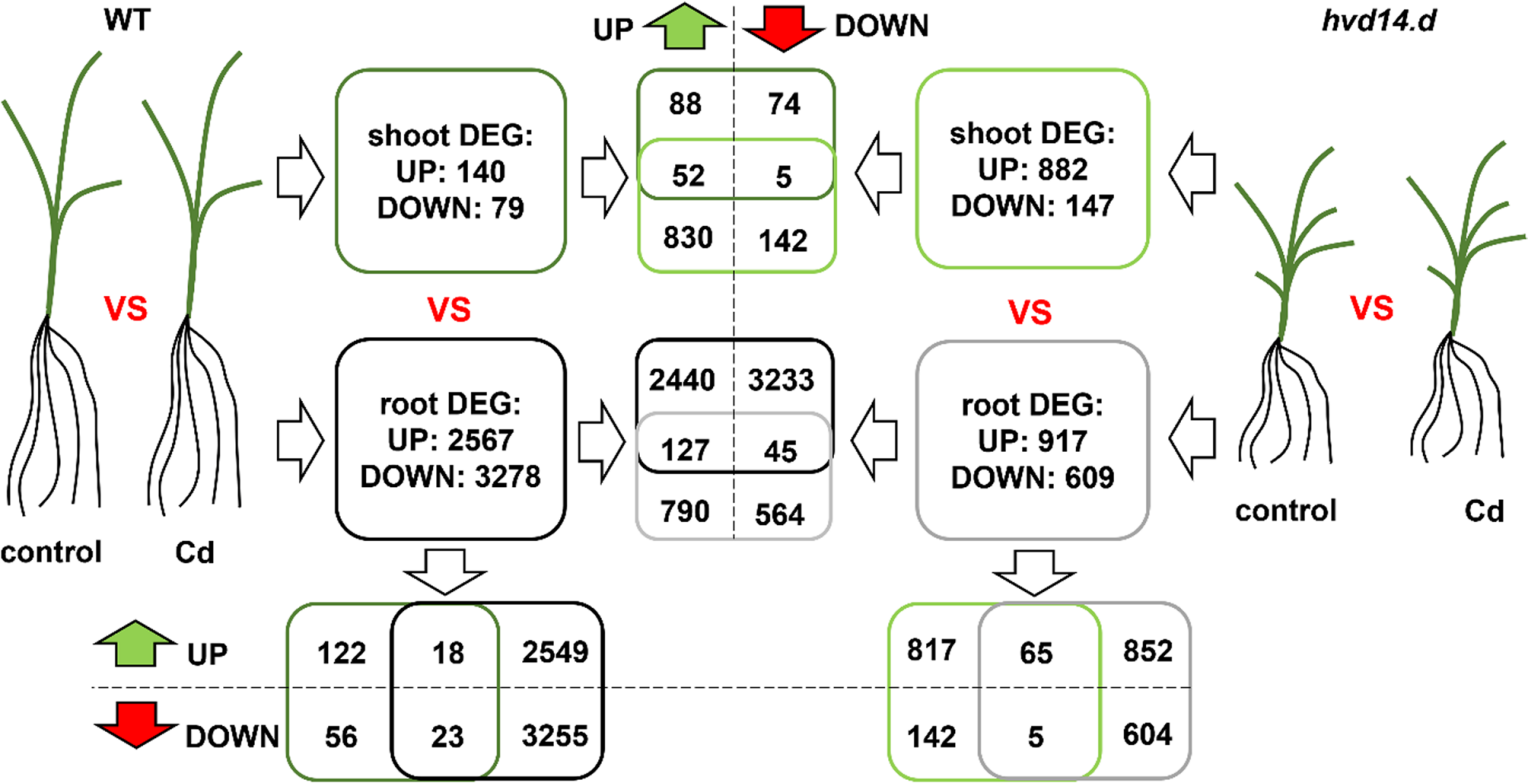
Transcriptomic response to Cd treatment in shoots and roots of WT and *hvd14.d* mutant barley plants. The number of DEGs(log₂FC ≥ 1 or ≤ − 1; adjusted *P* ≤ 0.01) identified in shoots and roots of control and Cd-treated plants is shown for both genotypes. Shared and genotype/tissue-specific DEGs in response to Cd were determined by comparing transcriptomic profiles between genotypes or tissues

In shoots of Cd-treated WT plants, 219 differentially expressed genes (DEGs; log2FC ≥ 1 or ≤ −1, adjusted P value ≤ 0.01) were identified (of which 140 were up- and 79 were down-regulated), whereas in WT roots, the treatment with Cd changed the expression of 5845 genes (2567 up- and 3278 down-regulated) (Fig. 2,Tab. S1). Among both sets of shoot and root DEGs, 18 up-regulated and 23 down-regulated genes were found to be common (Tab. S1). Under the same conditions in *hvd14.d*, the 1029 DEGs (882 up- and 147 down-regulated) in the shoot and 1526 DEGs (917 up- and 609 down-regulated) in the root were identified by comparison of the transcriptome of treated and control plants (Fig. 2, Tab. S2). There were 65 up- and 5 down-regulated common genes for the shoot and root of *hvd14.d* in response to Cd (Tab. S2).

Given that *hvd14.d* lacks functional SL perception, genes differentially expressed only in WT are likely to represent SL-dependent responses. To select genes involved in response to Cd treatment that are SL-dependent, the DEGs specific only for WT were identified. Comparison of Cd-responsive genes in the shoot revealed that only 57 genes were common for both genotypes (52 up- and 5 down-regulated), whereas the majority of DEGs were specific only for WT (162 genes; 88 up- and 74 down-regulated) or *hvd14* (972 genes; 830 up- and 142 down-regulated) (Tab. S3). Similar results were obtained when comparing the transcriptome of Cd-stressed roots of both genotypes: 172 (127 up- and 45 down-regulated) genes were common, 5673 genes were specific for WT (2440 up- and 3233 down-regulated), and 1354 genes were specific for *hvd14* (790 up- and 564 down-regulated) (Tab. S3). This strong genotype-specific transcriptional divergence further supports a key role of SL signaling in shaping the barley transcriptomic response to Cd stress.

Among the genes, the most up-regulated only in the WT shoot under Cd stress, a class III peroxidase (HORVU.MOREX.r2.7HG0537750.1) likely contributes to enhanced ROS detoxification [35]. A glucan endo-1,3-beta-glucosidase (HORVU.MOREX.r2.3HG0266100.1) may be involved in stress-induced cell wall remodeling [36]. Additionally, the induction of HAB1 (HORVU.MOREX.r2.1HG0077210.1), a regulator of ABA signaling, suggests a role for hormone-mediated stress adaptation [37] (Tab. S3). On the other hand, in WT roots under Cd stress, the HORVU.MOREX.r2.4HG0334890.1 was up-regulated. This gene, annotated as a Zn transporter, may contribute to Cd sequestration [38]. Additionally, HORVU.MOREX.r2.6HG0452050.1, described as a high-affinity nitrate transporter, might contribute to maintaining ion homeostasis, which can be disrupted under Cd toxicity [39].

Gene Ontology (GO) enrichment analysis of differentially expressed genes under Cd stress revealed that WT and *hvd14.d* mutant plants activate distinct biological pathways. Among up-regulated genes, WT shoots showed significant enrichment in processes such as defense response, and response to external biotic stimulus; all of which are associated with stress adaptation and detoxification mechanisms. In contrast, the *hvd14.d* mutant lacked enrichment in such canonical stress-related processes. While a few pathways linked to cell wall remodeling rather than direct detoxification were induced in WT. This deficiency in activating typical protective mechanisms agrees with the enhanced Cd sensitivity of the mutant (Fig. S2). The analysis of down-regulated genes further supports this conclusion. In WT shoots, suppression was noted in pathways such as cytosolic calcium (Cl) ion transport, cellular response to misfolded proteins, and response to endoplasmic reticulum stress, potentially reflecting a tightly regulated unfolded protein response under metal stress. WT roots showed broad suppression of pathways including defense response, and transmembrane transport, indicating an active reorganization of metabolic and signaling networks under Cd toxicity. In *hvd14.d* roots, however, down-regulated genes were enriched in critical stress-response pathways such as cellular oxidant detoxification, response to oxidative stress, and cellular response to toxic substance (Fig. S2). These indicate that core detoxification pathways are strongly repressed in the mutant, potentially impairing its capacity to handle oxidative and metal stress.

Finally, the principal component analysis (PCA) revealed a clear separation based on both tissue type and genotype in response to Cd treatment (Fig. S3). Shoot and root samples clustered distinctly along the first principal component (PC1), indicating strong transcriptomic divergence between tissues. Additionally, within each tissue type, samples of the mutant genotype formed separate clusters from WT plants, suggesting that SL signaling via HvD14 influences the transcriptomic response to Cd stress. The separation was especially pronounced in the root tissue, where *hvd14.d* samples diverged substantially from the WT group (Fig. S3).

Taken together, these findings demonstrate that the *hvd14.d* mutant fails to properly activate key defense and detoxification pathways and, simultaneously, downregulates essential stress-response functions, highlighting its compromised transcriptional response to Cd and explaining its heightened sensitivity to heavy metal exposure.

### SL-dependent transcriptional regulators modulating cd stress response

**Fig. 3:**
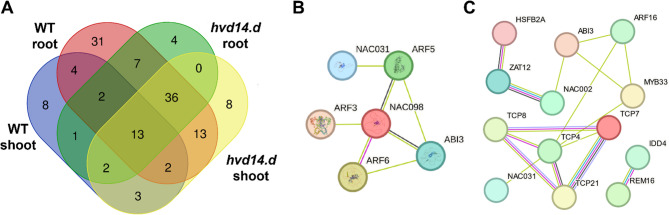
SL-dependent TFs controlling the Cd stress response in barley. (**A**) Venn diagram with the distribution of TFs predicted to regulate genotype- and tissue-specific DEGs in response to Cd treatment. (**B**) Interaction network of SL-related TFs identified for shoot-specific response in WT. (**C**) Interaction network of SL-related TFs identified for root-specific response in WT. The networks highlight candidate regulatory hubs potentially mediating SL-dependent control of the transcriptional response

To better understand the mechanisms underlying the distinct transcriptomic responses of WT and *hvd14.d* plants to Cd treatment, we focused on identifying TFs that could regulate the expression of DEGs in a tissue- and genotype-specific manner. First, promoter regions (−1500 bp) of all genotype-specific DEGs were screened for TF binding sites (Tab. S4). Next, TFs whose binding sites were overrepresented in the promoters of DEGs were identified, providing insight into regulatory factors with a potential key role in shaping the transcriptional response in shoot and root tissues of each genotype. In WT plants, 35 TFs were predicted to control the expression of 51.7% (150 out of 290) DEGs specific to the shoot, and 108 TFs were identified for the root, potentially regulating 72.9% (4262 out of 5848) root-specific DEGs. In the *hvd14.d* mutant, 65 TFs were predicted to control 85.8% (883 out of 1029) shoot-specific DEGs, and 77 TFs potentially regulated 76.9% (1174 out of 1526) root-specific DEGs. To uncover regulatory modules underlying tissue- and genotype-dependent responses, TFs were further classified as specific for WT or *hvd14.d* and/or tissue-specific (Tab. S4). These analyses revealed 43 TFs likely contributing to Cd response in WT plants (31 root-specific, 8 shoot-specific, and 4 common to both tissues) and 12 TFs regulating the transcriptomic response exclusively in *hvd14.d* (4 root-specific and 8 shoot-specific). Additionally, a group of 13 TFs was found to regulate Cd responses in both genotypes and in both tissues; these factors may represent SL-independent regulatory components (Fig. 3A). For each genotype-specific TF, homologs in *Arabidopsis thaliana* were identified (Tab. S5). Several of these homologs are known regulators of heavy metal responses, including AT5G59780 (MYB59) [40], AT2G27050 (EIL1), and AT1G73730 (EIL3) [41, 42]. Moreover, SL-related TFs contributing to the barley response to Cd were used to construct interaction networks for both shoot (Fig. 3B) and root tissues (Fig. 3C), highlighting candidate regulatory hubs that may mediate SL-dependent control of stress-responsive transcription.

### Barley transcriptome response to Zn treatment

**Fig. 4:**
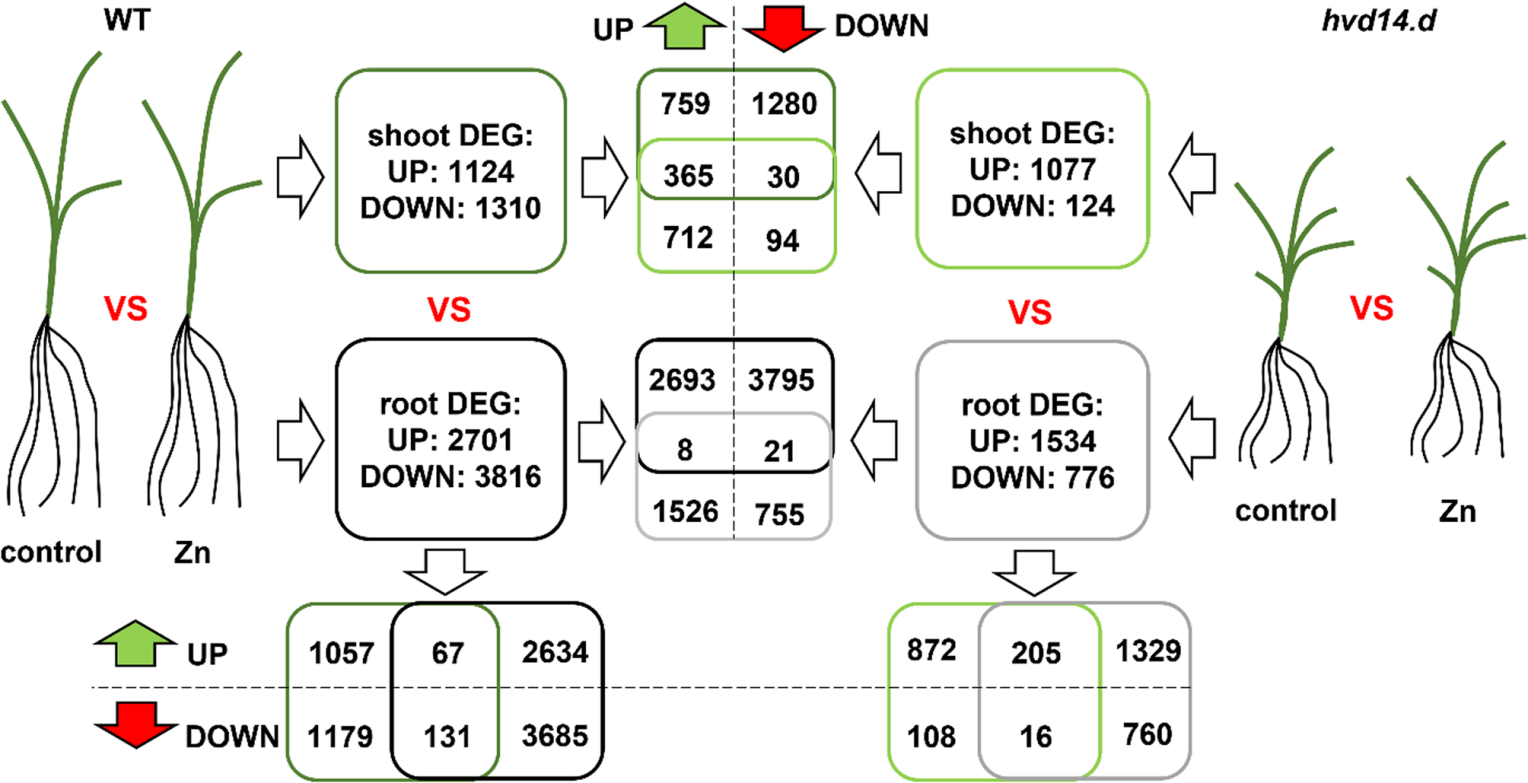
Transcriptomic response to Zn treatment in shoots and roots of WT and ***hvd14.d*** mutant barley plants. The number of DEGs (log₂FC ≥ 1 or ≤ − 1; adjusted *P* ≤ 0.01) identified in shoots and roots of control and Zn-treated plants is shown for both genotypes. Shared and genotype/tissue-specific DEGs in response to Zn were determined by comparing transcriptomic profiles between genotypes or tissues

For the data obtained from the Zn treatment, the same strategy as described above was applied. This approach enabled the identification of 2434 DEGs in the WT shoot (1124 up- and 1310 down-regulated) and 6517 DEGs in the WT root (2701 up- and 3816 down-regulated) (Fig. 4), Tab. S6). Among both sets of shoot and root DEGs, the common 67 up- and 131 down-regulated genes were found in WT (Tab. S6). On the other hand, *hvd14.d* responds to Zn treatment by changing the expression of 1201 (1077 up- and 124 down-regulated) and 2310 (1534 up- and 776 down-regulated) genes in the shoot and root, respectively (Fig. 4), Tab. S7). Among DEGs in the shoot and root of *hvd14.d*, 221 (205 up- and 16 down-regulated) common genes were identified (Tab. S7). Comparison of transcriptome response of WT and *hvd14.d* to Zn allowed the selection of SL-dependent genes whose expression was affected by treatment. We identified 2039 DEGs in the shoot (759 up- and 1280 down-regulated) and 6488 DEGs in the root (2693 up- and 3795 down-regulated), specific only for WT, thus related to the SL signal perception. On the other hand, 806 DEGs in the shoot (712 up- and 94 down-regulated) and 2281 DEGs in the root (1526 up- and 755 down-regulated) were identified as specific only for the *hvd14.d* mutant. Finally, 395 and 29 genes were common for both genotypes in the shoot and root, respectively (Fig. 4, Tab. S8). Among the genes specifically up-regulated in WT shoot under Zn stress, a glutathione S-transferase (HORVU.MOREX.r2.5HG0429310.1) likely contributes to Zn detoxification through glutathione conjugation [43]. Two cysteine-rich receptor-like kinases (HORVU.MOREX.r2.5HG0435700.1 and HORVU.MOREX.r2.2HG0114500.1) may participate in redox-dependent stress signaling triggered by zinc exposure [44]. Additionally, a BRCA1-associated RING domain protein (HORVU.MOREX.r2.2HG0150020.1) may be involved in stress response regulation via ubiquitin-mediated pathways and ROS production [45]. Finally, a UDP-galactose/UDP-glucose 4-epimerase (HORVU.MOREX.r2.4HG0344600.1) might support cell wall remodeling or sugar metabolism adjustments under Zn-induced stress [46] (Tab. S8). Among the genes up-regulated specifically in WT root under Zn stress, several candidates are involved in redox homeostasis and metal-related stress response. A NADH-ubiquinone oxidoreductase (HORVU.MOREX.r2.7HG0598160.1) may support mitochondrial electron transport and mitigate Zn-induced oxidative stress [47]. The induction of laccase-10 (HORVU.MOREX.r2.4HG0323160.1), an enzyme associated with oxidative polymerization and potentially involved in lignin biosynthesis, suggests reinforcement of cell wall structures in response to metal exposure [48]. Additionally, HORVU.MOREX.r2.5HG0399100.1, encoding the MARD1 (MEDIATOR OF ABA-REGULATED DORMANCY1) protein, may be linked to ABA- and redox-mediated stress signaling [49] (Tab. S8).

The GO enrichment analysis revealed clear differences in the transcriptional response patterns between the two genotypes. In WT plants, up-regulated genes in both shoots and roots showed strong enrichment for biological processes related to ribosome biogenesis, rRNA processing, and protein translation. In particular, processes such as maturation of LSU-rRNA, ribosomal large subunit biogenesis, and ribosome assembly were significantly overrepresented (Fig. S4). These processes are often associated with cellular recovery processes, suggesting that WT plants can reprogram protein synthesis pathways to mitigate the damage associated with excess Zn. Additionally, WT roots showed activation of more specific stress-responsive categories such as response to water deprivation, response to acid chemical, and response to biotic stimulus, indicating a broad-spectrum protective transcriptional program likely contributing to stress tolerance. In contrast, the *hvd14.d* mutant exhibited a markedly different transcriptional landscape. Although some enrichment of ribosome-related processes was still observed, the magnitude and diversity of activated GO terms were reduced. Moreover, many stress-adaptive pathways that were enriched in WT, such as ion transport, response to abiotic stimulus, and detoxification-related processes, were either absent or significantly diminished in the mutant (Fig. S4). This discrepancy was especially pronounced in the root tissue, where the mutant failed to induce key components of the transcriptional program associated with Zn detoxification and ion homeostasis. Such a failure may result from impaired signaling capacity, possibly linked to disrupted SL-mediated regulation. The GO enrichment profiles suggest that the *hvd14.d* mutant exhibits a suboptimal and narrower transcriptional response to Zn, particularly lacking coordinated induction of detoxification and homeostasis-related pathways. This may underlie the increased sensitivity of the mutant to Zn stress, resulting from insufficient activation of the protective and compensatory mechanisms observed in the WT.

PCA of RNA-seq data revealed a clear separation of samples based on tissue type along PC1, which explained 45.7% of the total variance. Root and shoot samples clustered distinctly, indicating a strong tissue-specific transcriptional response to Zn exposure. Within each tissue, additional separation was observed between genotypes. Notably, *hvd14.d* root samples diverged strongly from WT root samples along PC2 (11.4% of the variance), suggesting a pronounced genotype-specific response to Zn in the root. In contrast, the differences between WT and *hvd14.d* shoots were more subtle, implying that the mutation has a more substantial impact on root transcriptomes under Zn stress (Fig. S5).

All analyses consistently indicate that WT and *hvd14.d* differ in their transcriptomic response to Zn stress. WT plants activated a broader and more coordinated set of stress-responsive genes, while the *hvd14.d* mutant exhibited a weaker and less diverse response. These differences were particularly evident in the root tissue and were reflected in both the number of DEGs and the functional enrichment profiles. PCA further confirmed genotype-specific transcriptional patterns. Collectively, the data suggest that *hvd14.d* is more sensitive to Zn, possibly as a result of insufficient activation of the protective pathway.

### SL-dependent transcriptional regulators modulating Zn stress response

**Fig. 5:**
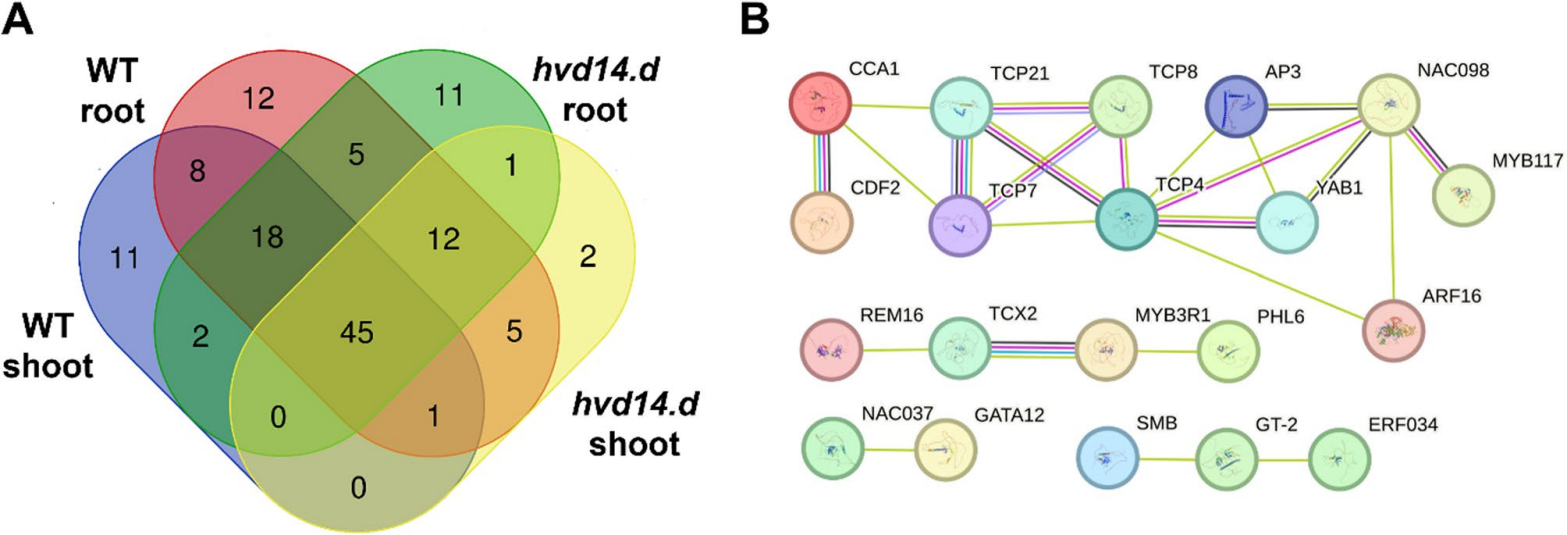
SL-dependent TFs controlling the Zn stress response in barley. (**A**) Venn diagram showing the distribution of TFs predicted to regulate genotype- and tissue-specific DEGs in response to Zn treatment. (**B**) Interaction network of SL-related TFs identified for shoot-specific response in WT. The network highlights candidate regulatory hubs potentially mediating SL-dependent control of Zn-responsive transcription

Analyses of the promoter regions of DEGs specific to both genotypes (Tab. S9) revealed the TFs that regulate the barley response to Zn in both SL-dependent and SL-independent manners, based on binding site enrichment analysis (Tab. S10). In total, 31 overrepresented TFs that regulate the Zn response in WT were identified (11 shoot-specific, 12 root-specific, 8 common for both tissues). On the other hand, only 14 overrepresented TFs that mediate transcriptome response to Zn in *hvd14.d* were identified (2 shoot-specific, 11 root-specific and 1 common for both tissues) (Fig. 5A). For each genotype-specific TF, the corresponding Arabidopsis homologs were identified (Tab. S10), including AT1G79580 (SMB) involved in stress response [50], AT1G34370 (STOP1) [51] and AT5G22890 (STOP2) [52] involved in H^+^ and Al^3+^ toxicity in root and shoot (Tab. S10). In addition, interaction networks related to SL-associated TFs implicated in the barley response to Zn were identified for the shoot (Fig. 5B).

## Discussion

Our results demonstrate that the barley *hvd14.d* mutant, impaired in SL signaling, is more sensitive to Cd or Zn stress than WT. These findings support previous bioinformatic predictions of co-expression networks linking SL biosynthesis genes to heavy metal stress responses in rice [20]. Our physiological, ionomic and transcriptomic data are consistent with these predictions, revealing that *hvd14.d* exhibited stronger growth inhibition, disrupted nutrient homeostasis, and photosynthetic damage under metal stress, compared to the WT.

### SL deficiency exacerbates metal toxicity and alters metal accumulation

The *hvd14.d* mutant accumulated significantly more Cd in both shoots and roots than the WT (Table 1), which is in line with studies showing that SLs limit Cd uptake and translocation. For instance, in *A. annua*, SL application reduced Cd accumulation by 39–56% [24], while in soybean, SLs downregulated Cd transporters (e.g., heavy metal ATPase, HMA) to restrict root-to-shoot transfer [26]. Conversely, the mutant’s reduced shoot Zn levels, paired with root Zn hyperaccumulation, suggest SLs regulate Zn partitioning, possibly via HMA transporters, as observed in wheat [25]. Impaired micronutrient homeostasis in *hvd14.d* (e.g., reduced Mn and Fe under Cd stress) further mirrors the SL-mediated metal crosstalk previously reported, where SLs enhance Fe uptake under Cd stress [17].

Although altered Cd and Zn accumulation was evident in *hvd14.d*, only a few transporters appeared among the DEGs, such as *HORVU.MOREX.r2.4HG0334890.1* (Zn transporter) and *HORVU.MOREX.r2.6HG0452050.1* (nitrate transporter). The absence of broader transcriptional changes does not preclude functional regulation, since transporter activity can be controlled post-transcriptionally or post-translationally (e.g. via protein stability, phosphorylation, or subcellular localization). Thus, SL signaling may affect metal uptake and partitioning through mechanisms beyond gene expression.

Besides Cd and Zn, our ionomic analysis also revealed genotype-dependent changes in the accumulation of other essential elements (Table 1). For example, Cd stress reduced Ca and Fe in WT leaves, while *hvd14.d* displayed altered Ca and Mg partitioning between shoots and roots. Both Cd and Zn led to decreased Mn accumulation in roots, with a stronger reduction observed in the mutant. These shifts indicate that impaired SL perception not only affects Cd and Zn handling but also influences broader ion homeostasis, potentially by altering ion selectivity or competition for transport pathways. Thus, HvD14-mediated SL signaling may contribute to maintaining the balance of multiple nutrients under heavy metal stress.

### SLs protect photosynthesis and redox balance

The *hvd14.d* mutant exhibited severe chlorophyll loss and PSII photoinhibition under Cd/Zn stress (Fig. 1; Table 2), contrasting with the resilience of WT. This is consistent with studies showing that SLs preserved chloroplast ultrastructure and upregulated expression of genes encoding psbA/psbB components in *A. annua* [24] and mitigated Zn-induced PSII damage in rape [53]. Moreover, in response to drought, *hvd14.d* also exhibited stronger chloroplast disorganisation and impaired photosynthesis, compared to WT [15]. A positive effect of synthetic SL treatment on photosynthetic parameters in rice [54] and rapeseed (*Brassica napus* L.) [55] under salt stress was demonstrated. These findings collectively suggest that SL signalling plays an important role in maintaining photosynthesis under stress conditions.

Given that photosynthetic impairment is closely linked to oxidative stress under heavy metal exposure, we next analyzed ROS-related responses. Anthocyanin accumulation, the effect of oxidative stress, observed in mutant (Table 2), further supports the role of SLs in ROS scavenging, consistent with GR24-induced superoxide dismutase (SOD)/catalase (CAT) activation in wheat [25]. On the other hand, the role of SLs in ROS scavenging was previously described in various species [18], including barley [16, 17]. The drought-induced accumulation of hydrogen peroxide (H_2_O_2_) present in the *hvd14.d* leaf tissue was significantly higher than in the WT leaves [16], whereas in barley seedlings subjected to Cd stress, an increased level of H_2_O_2_ was detected, which was decreased after synthetic SLs application [17]. Among genes induced exclusively in WT under Cd stress, we identified candidates with previously postulated roles in metal stress responses, although not previously linked to SL signaling. The highest level of Cd-induced expression in the WT shoot was observed for the gene HORVU.MOREX.r2.7HG0537750, encoding a member of class III peroxidase involved in ROS scavenging and cell wall remodeling [56] (Tab. S1). It was previously shown that peroxidases play a key role in Cd stress response in Arabidopsis [57] and rice (*Oryza sativa*) [58] enhancing Cd tolerance by reducing oxidative damage. A less effective ROS neutralization system in the mutant results in higher toxicity of Cd and Zn treatment. Importantly, ROS production and scavenging are also regulated by ABA [59, 60].

### Reduced sensitivity of *hvd14.d* to ABA results in enhanced susceptibility to cd or Zn stress

It has been previously shown that hvd14.d exhibits reduced sensitivity to ABA under both control and drought conditions. Whereas 300 µM ABA almost completely inhibited seed germination in WT, mutant seeds maintained a germination capacity of approximately 70% [15]. Additionally, the increased sensitivity of hvd14.d to drought was associated with a weaker response to drought and a lack of activation of ABA-related mechanisms, resulting in slower stomatal closure, faster water loss, weaker wax deposition, and impaired photosynthesis compared to the WT [16]. The previously described aspects of increased sensitivity to Cd or Zn stress may be related to the impairment of ABA signaling pathways in the mutant. It has been demonstrated that ABA, by controlling stomatal closure and transpiration rates, regulates the translocation of heavy metals from roots to shoots [61, 62]. In Arabidopsis, ABA treatment decreased Cd uptake by suppressing the activity of the iron-regulated transporter 1 (IRT1), thereby alleviating the toxic effects of Cd [63, 64]. On the other hand, treatment of *Vitis vinifera* L. seedlings with 10 µM ABA alleviated Zn stress by lowering Zn absorption and accumulation in the roots, while simultaneously enhancing the expression of ZIP and other detoxification-associated genes in both roots and leaves [65]. Hence, slower stomata closure in the mutant may result in the accumulation of Cd or Zn in tissues and a faster achievement of toxic concentrations (Tables 1 and 2).

### SL-dependent transcriptomic modulation under cd or Zn stress

Our data shows that *hvd14.d* exhibits the altered transcriptomic response to Cd/Zn stress compared to WT (Figs. 2 and 4). While WT roots and shoots activated numerous genes involved in detoxification, ion homeostasis, and hormone signaling, including ABA regulators (Fig. S2 and S4), *hvd14.d* failed to activate similar defense mechanisms. Comparison of the transcriptomes of WT and *hvd14.d* during the response to Cd/Zn treatment enabled the identification of SL-dependent genes involved in this process (Tab. S3 and S8). Among these DEGs, several ABA-induced genes were found, supporting previous findings that indicated reduced ABA sensitivity in the *hvd14.d* mutant. Additionally, in both Cd or Zn treatments, genes associated with detoxification, metal transport, ROS management, and general stress responses were represented (Tab. S3 and S8). Genes involved in detoxification were strongly induced in WT under both stress conditions. Further analysis of individual genes identified as SL-dependent provide new insights into the role of SLs in plant responses to heavy metals and to the characterization of the molecular mechanisms underlying barley response to this stress.

### SL-dependent TFs regulating barley response to cd and Zn stress

**Fig. 6:**
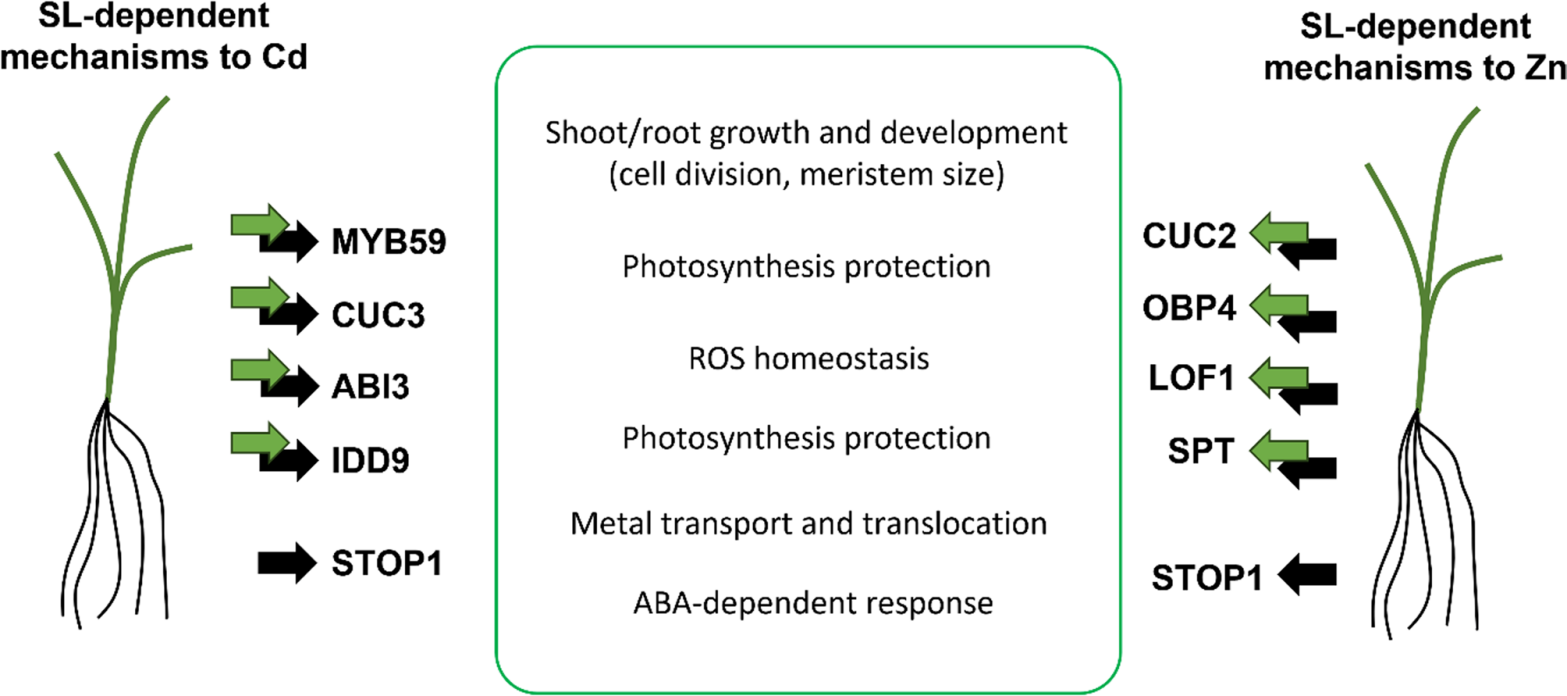
SL-dependent TFs controlling barley responses to Cd or Zn stress. Summary of key SL-dependent TFs predicted to regulate shoot- and root-specific responses to Cd (left) and Zn (right) stress in WT plants. Green arrows indicate TFs active in the shoot; black arrows indicate TFs active in the root. The middle panel summarizes the main biological processes regulated by these TFs, contributing to plant adaptation to metal stress

To gain insight into how SLs might influence gene expression in response to heavy metals, we analyzed the DEGs specific to the WT plants to identify TFs that could be regulating these genes (Tab. S5 and S10). In both Cd and Zn treatments, this approach allowed us to identify TFs that were commonly active in both shoot and root tissues, highlighting shared regulatory components involved in the plant’s response to each type of metal stress. In the case of Cd treatment, four TFs were identified as commonly regulated in both shoot and root (MLOC_52439, MLOC_13932, MLOC_69727, and MLOC_1876) (Tab. S5). Analysis of their Arabidopsis homologs suggested that these genes may encode MYB59, CUP-SHAPED COTYLEDON3 (CUC3), ABSCISIC ACID INSENSITIVE3 (ABI3), and INDETERMINATE-DOMAIN9 (IDD9), respectively, all of which are known to be involved in the regulation of stress responses and developmental processes. It was previously shown that MYB59 is induced by Cd in Arabidopsis and acts as a negative regulator of Ca signaling and homeostasis [40]. Moreover, it was proposed that MYB59 expression varies across metallicolous and non-metallicolous populations of *Arabidopsis halleri*, reflecting distinct strategies of Cd tolerance and accumulation [66]. CUC3 TF was originally identified as involved in boundary and shoot meristem formation in Arabidopsis [67], but its role in controlling root development in response to manganese deficiency was later proposed [68]. Thus, it can be hypothesized that this TF may play a key role in shaping both shoot and root system architecture in response to stress conditions in an SL-dependent manner. On the other hand, ABI3 is known to play a key role in the establishment of seed dormancy [69], but it was also postulated that this TF might function as a general regulator for the timing of developmental transitions throughout the life cycle of plants [70]. Additionally, its role in response to drought conditions has been proven [71], and abi3 mutants have been shown to exhibit increased sensitivity to Cd treatment during germination [72]. Considering previous results indicating the involvement of ABA-dependent mechanisms in the WT response to Cd stress, this TF may play a key role in coordinating these processes in shoot and root tissues. Finally, the IDD9 is involved in root development [73] and its expression is induced by H_2_O_2_ [74], suggesting that it may contribute to signal transduction related to ROS (Fig. 6).

Analysis of SL-dependent transcription regulatory network in response to Zn allowed the identification of eight key TFs active in shoot and root (Tab. S10). The roles of four of them, AT4G33280, AT3G13040, AT1G76890, and AT4G30080, remain unknown. However, the other four: CUP-SHAPED COTYLEDON2 (CUC2), OBF BINDING PROTEIN 4 (OBP4), LATERAL ORGAN FUSION1 (LOF1), SPATULA (SPT) were previously functionally characterised. CUC2 action is mainly linked with cytokinin-dependent developmental processes, such as floral development [75, 76]. Additionally, a role of CUC2 in shoot branching [77] and in ABA-related responses to various stresses has also been proposed [78]. These data suggest that CUC2 may be utilized by SLs to regulate shoot and root architecture, as well as to activate defense mechanisms in response to Zn stress. OBF4 was characterized as a negative regulator of cell proliferation and expansion in Arabidopsis [79], which plays a role in the modulation of root system architecture [80, 81] as well as response to salt stress [82]. Thus, this TF may be involved in the reprogramming of shoot and root growth in response to Zn. A similar function is proposed for LOF1, which is involved in lateral organ separation and axillary meristem formation in Arabidopsis [83]. The last TF, SPT was described as the regulator of cotyledon expansion [84] and affects root development via regulation of root meristem size [85]. Its identification in this work suggests that SPT might participate in yet uncharacterized aspects of the Zn stress response, possibly in an SL-dependent manner (Fig. 6).

Interestingly, despite the differences between Cd or Zn and the distinct plant responses to these metals, it was possible to identify common SL-dependent TFs involved in mediating plant adaptation to both treatments. Among them, the most interesting is STOP1, active in the WT root under Cd/Zn treatment. STOP1 is known to regulate multiple genes that protect Arabidopsis from proton and aluminium (Al^3+^) toxicities, mainly by organic acid exudation and cellular detoxification [86]. Its activation in response to Cd and Zn suggests that STOP1 may function as a more general regulator of metal stress responses, potentially coordinating defense mechanisms beyond those specific to aluminium. The presence of STOP1 activity in barley roots under both treatments also raises the possibility that SLs may influence its expression or activity, linking SL signaling with key components of the transcriptional network regulating heavy metal tolerance.

Our study is not without limitations. The conclusions are drawn from a single, however well-characterized *hvd14.d* mutant, and we did not attempt genetic complementation or SL treatment. Moreover, we did not quantify endogenous hormone levels under stress. While these aspects remain open to future research, the combination of detailed physiological, ionomic, and transcriptomic data consistently suggests a central role of SL perception in shaping barley responses to Cd and Zn. By providing the first integrative view of HvD14 function under heavy metal stress, we offer a framework that can now be expanded by additional genetic and biochemical approaches.

## Conclusions

This study provides the first experimental evidence that endogenous SL perception modulates barley tolerance to Cd and Zn stress. Impairment of SL signaling in the *hvd14.d* mutant resulted in increased metal accumulation, disturbed nutrient balance, and reduced capacity to counteract oxidative damage. Comparative transcriptomic analyses revealed that SLs shape the activation of gene networks associated with detoxification, metal transport, redox regulation, and key TFs mediating stress adaptation. The results further indicate an interplay between SL signaling and ABA-dependent pathways in controlling metal uptake and oxidative stress responses. Importantly, SL-dependent TFs emerged as candidates for the regulation of adaptive mechanisms under heavy metal stress. Together, these findings position SLs as central coordinators of transcriptional and physiological adjustments in both shoots and roots exposed to Cd and Zn. While future work with complementary genetic resources and hormone profiling will refine the mechanistic details, the present study establishes HvD14 as a critical regulatory node linking hormonal signaling with ion homeostasis and stress tolerance in barley.

## Supplementary Information

Supplementary Material 1. Supplementary Table 1. DEGs in WT barley plants in response to Cd treatment

Supplementary Material 2. Supplementary Table 2.DEGs in *hvd14.d* barley plants in response to Cd treatment.

Supplementary Material 3. Supplementary Table 3. Genotype-specific and shared DEGs in shoot and root of WT and *hvd14.d* barley in response to Cd treatment.

Supplementary Material 4. Supplementary Table 4. TFs and their binding site prediction in promoter regions of Cd-responsive genes in WT and *hvd14.d* barley 

Supplementary Material 5. Supplementary Table 5. TFs that regulate barley response to Cd in SL-dependent and SL-independent manner

Supplementary Material 6. Supplementary Table 6.DEGs in WT barley plants in response to Zn treatment

DEGs in *hvd14.d* barley plants in response to Zn treatment.

Supplementary Material 8. Supplementary Table 8. Genotype-specific and shared DEGs in shoot and root of WT and *hvd14.d* barley in response to Zn treatment.

Supplementary Material 9. Supplementary Table 9. TFs and their binding site prediction in promoter regions of Zn-responsive genes in WT and *hvd14.d* barley

Supplementary Material 10. Supplementary Table 10. TFs that regulate barley response to Zn in SL-dependent and SL-independent manner

Supplementary Material 11. Fig. S1. Phenotypic response of wild-type (WT) and *hvd14.d* mutant barley plants to cadmium (Cd) and zinc (Zn) stress. Representative images of WT and *hvd14.d* plants grown under control conditions or subjected to 5 µM Cd or 50 µM Zn treatments. Scale bars = 10 cm. Fig. S2. Gene Ontology enrichment analysis of Cd-responsive genes in shoot and root tissues of WT and *hvd14.d* barley plants. Bar plots display GO terms significantly enriched (Fold Enrichment >1) among up-regulated (left panels) and down-regulated (right panels) genes in shoots (top panels) and roots (bottom panels) of WT (orange) and *hvd14.d* (red) plants treated with Cd. Each bar represents a single GO biological process term, and its length corresponds to the fold enrichment value. Differences in the enriched categories reflect distinct transcriptional responses to Cd in WT and *hvd14.d*, with the mutant showing a stronger activation of processes related to detoxification and oxidative stress in roots. Fig. S3. Principal component analysis (PCA) of gene expression profiles in barley under Cd treatment. PCA was performed on log-transformed and z-score normalized FPKM values from RNA-seq data of WT and hvd14.d mutant plants treated with Cd. Four biological replicates were analyzed for each combination of genotype (WT, hvd14.d) and tissue (root, shoot). PC1 and PC2 explain 46.0% and 9.6% of the total variance, respectively. Genotypes are color-coded (green for WT, orange for hvd14.d), and tissue types are indicated by marker shape (circle for shoot, cross for root). Fig. S4. Gene Ontology enrichment analysis of Zn-responsive genes in shoot and root tissues of WT and *hvd14.d* barley plants. Bar plots illustrate significantly enriched biological processes (Fold Enrichment >1) among up-regulated (left panels) and down-regulated (right panels) genes in the shoots (top panels) and roots (bottom panels) of WT (orange) and *hvd14.d* (red) plants exposed to Zn. The enriched GO terms reflect genotype-specific responses to Zn stress, with *hvd14.d* showing a stronger activation of signaling, detoxification, and ion transport processes, particularly in the root tissue. Fig. S5. Principal component analysis (PCA) of gene expression profiles in barley under Zn treatment.PCA was performed on log-transformed and z-score normalized FPKM values from RNA-seq data of WT and *hvd14.d* mutant plants treated with Zn. Four biological replicates were analyzed for each combination of genotype (WT, *hvd14.d*) and tissue (root, shoot). PC1 and PC2 explain 45.7% and 11.4% of the total variance, respectively. Genotypes are color-coded (green for WT, orange for *hvd14.d*), and tissue types are indicated by marker shape (circle for shoot, cross for root).

## Data Availability

Transcriptomic data: ArrayExpress repository E-MTAB-13641 (https://www.ebi.ac.uk/biostudies/arrayexpress/studies/E-MTAB-13641; control conditions) and E-MTAB-15240 (https://www.ebi.ac.uk/biostudies/arrayexpress/studies/E-MTAB-15240, Cd and Zn treatment). Plant material will be made available on request. https://www.ebi.ac.uk/biostudies/arrayexpress/studies/E-MTAB-15240?key=8523fe09-8f28-4b4c-8766-792ef48bee75.
