# Supplementary material for: Dissecting the role of strigolactone perception in barley tolerance to cadmium or zinc stress based on the receptor mutant analysis
Source: BMC Plant Biol. 2025 Nov 21;25:1618. doi: 10.1186/s12870-025-07683-4 (PMC12639677; doi:10.1186/s12870-025-07683-4)
Supplement: Supplementary file 11 — Supplementary Material 11. Fig. S1. Phenotypic response of wild-type (WT) and hvd14.d mutant barley plants to cadmium (Cd) and zinc (Zn) stress. Representative images of WT and hvd14.d plants grown under control conditions or subjected to 5 µM Cd or 50 µM Zn treatments. Scale bars = 10 cm. Fig. S2. Gene Ontology enrichment analysis of Cd-responsive genes in shoot and root tissues of WT and hvd14.d barley plants. Bar plots display GO terms significantly enriched (Fold Enrichment >1) among up-regulated (left panels) and down-regulated (right panels) genes in shoots (top panels) and roots (bottom panels) of WT (orange) and hvd14.d (red) plants treated with Cd. Each bar represents a single GO biological process term, and its length corresponds to the fold enrichment value. Differences in the enriched categories reflect distinct transcriptional responses to Cd in WT and hvd14.d, with the mutant showing a stronger activation of processes related to detoxification and oxidative stress in roots. Fig. S3. Principal component analysis (PCA) of gene expression profiles in barley under Cd treatment. PCA was performed on log-transformed and z-score normalized FPKM values from RNA-seq data of WT and hvd14.d mutant plants treated with Cd. Four biological replicates were analyzed for each combination of genotype (WT, hvd14.d) and tissue (root, shoot). PC1 and PC2 explain 46.0% and 9.6% of the total variance, respectively. Genotypes are color-coded (green for WT, orange for hvd14.d), and tissue types are indicated by marker shape (circle for shoot, cross for root). Fig. S4. Gene Ontology enrichment analysis of Zn-responsive genes in shoot and root tissues of WT and hvd14.d barley plants. Bar plots illustrate significantly enriched biological processes (Fold Enrichment >1) among up-regulated (left panels) and down-regulated (right panels) genes in the shoots (top panels) and roots (bottom panels) of WT (orange) and hvd14.d (red) plants exposed to Zn. The enriched GO terms reflect gen [file 12870_2025_7683_MOESM11_ESM.pptx]

## Slide 1
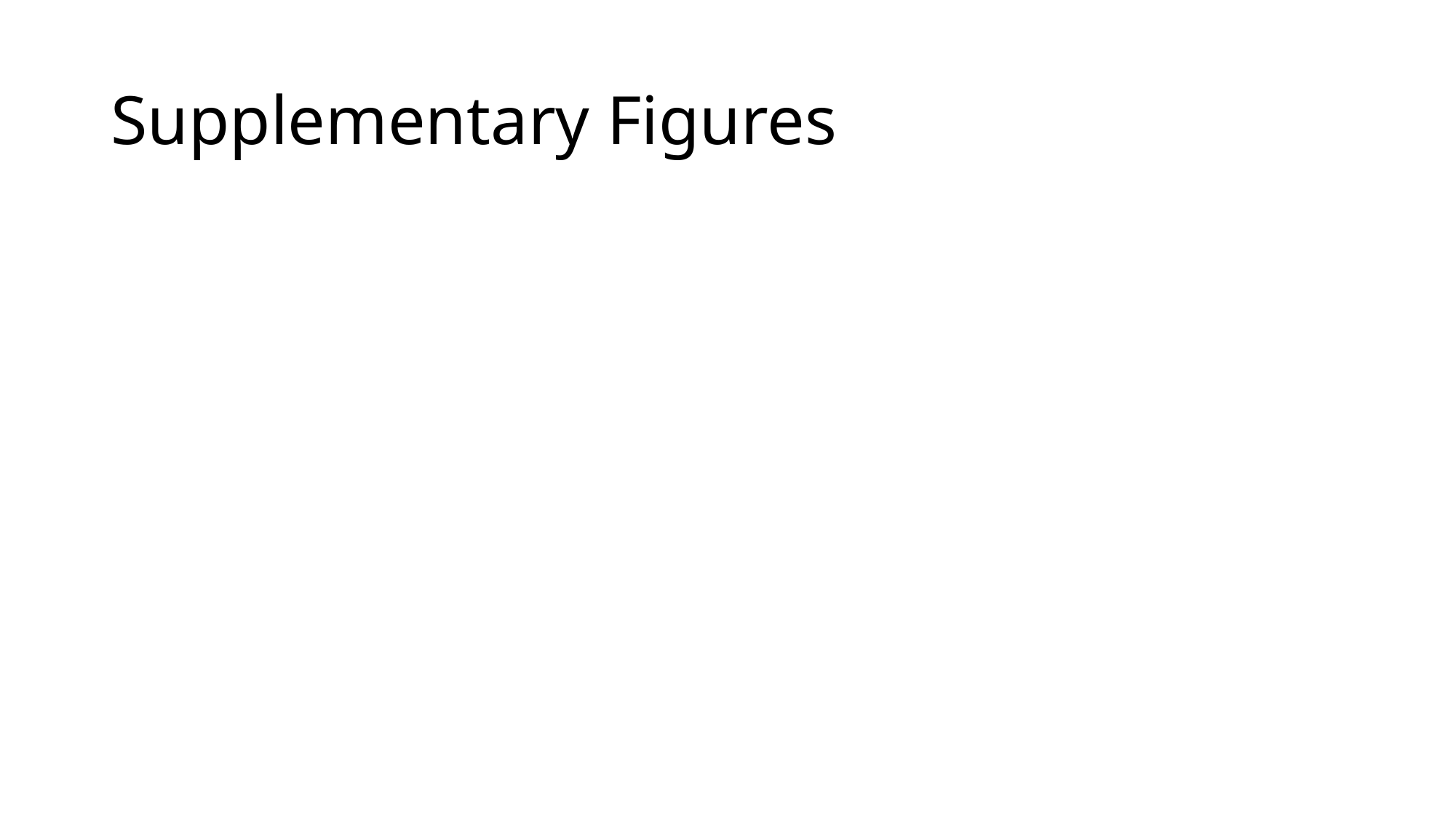

# Supplementary Figures

## Slide 2
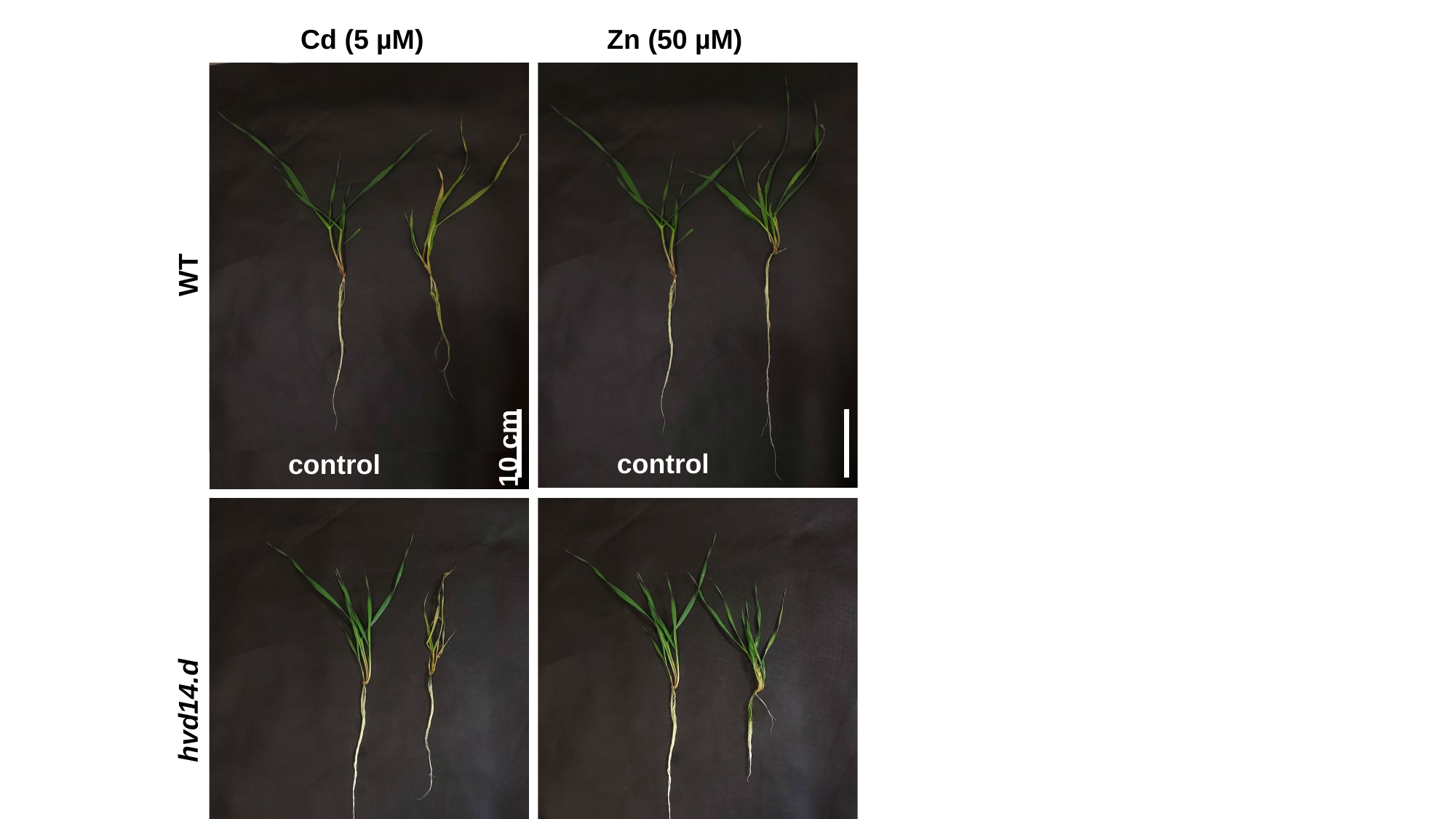

# Fig. S1
Zn (50 µM)
Cd (5 µM)
WT
10 cm
control
control
hvd14.d
control
control

## Slide 3
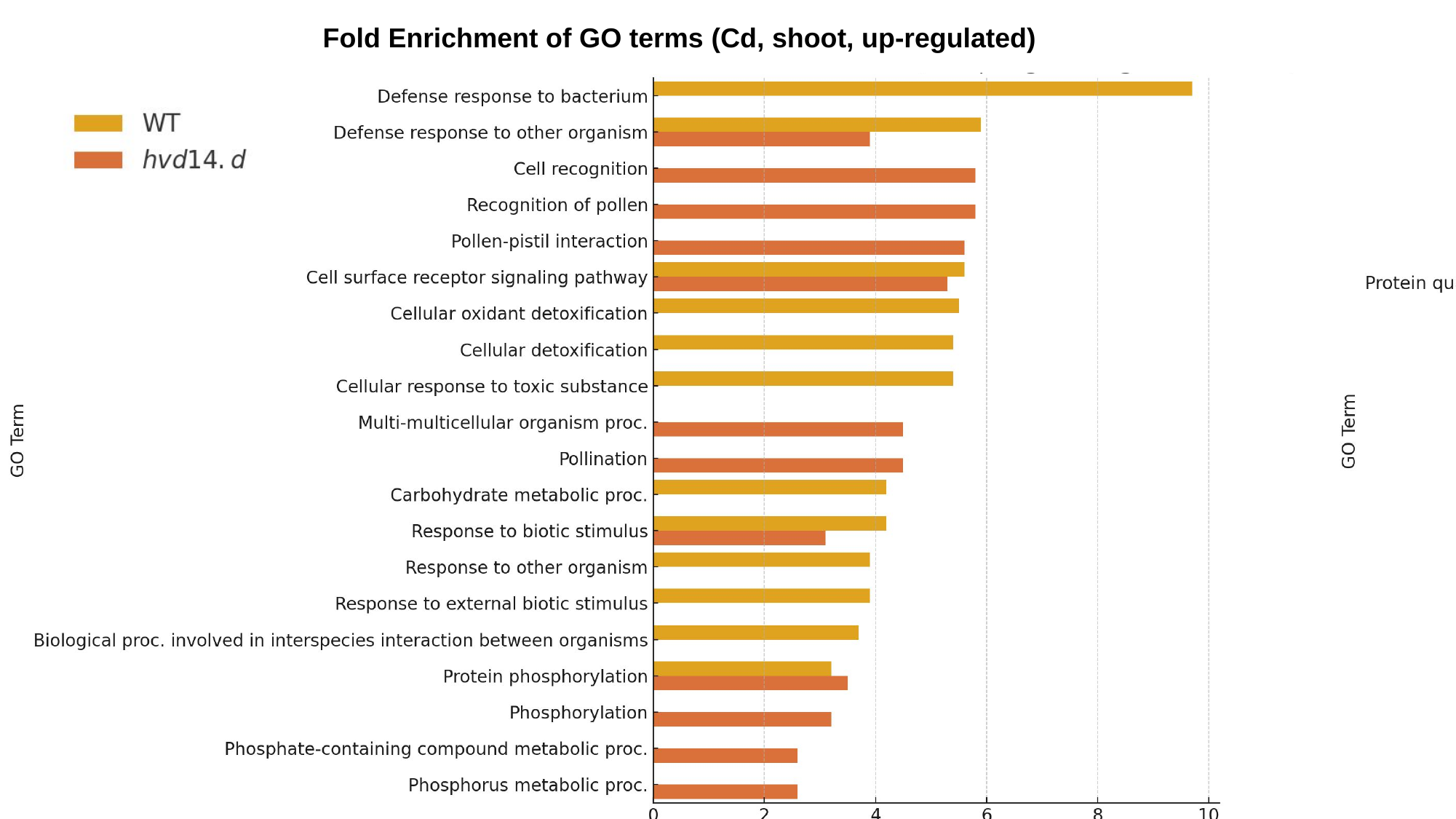

# Fig. S2
Fold Enrichment of GO terms (Cd, shoot, down-regulated)
Fold Enrichment of GO terms (Cd, shoot, up-regulated)
Fold Enrichment of GO terms (Cd, root, down-regulated)
Fold Enrichment of GO terms (Cd, root, up-regulated)

## Slide 4
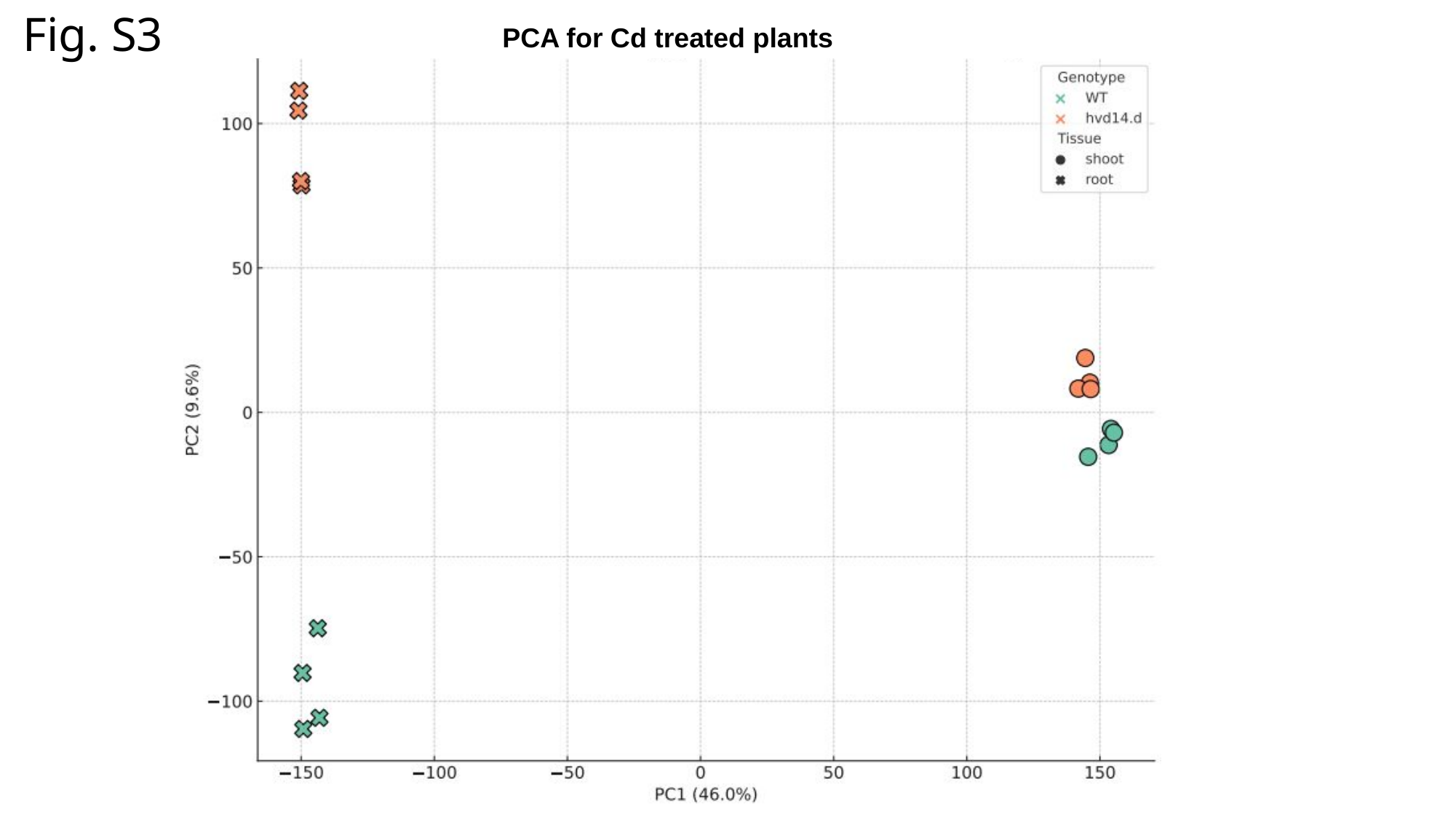

Fig. S3
PCA for Cd treated plants

## Slide 5
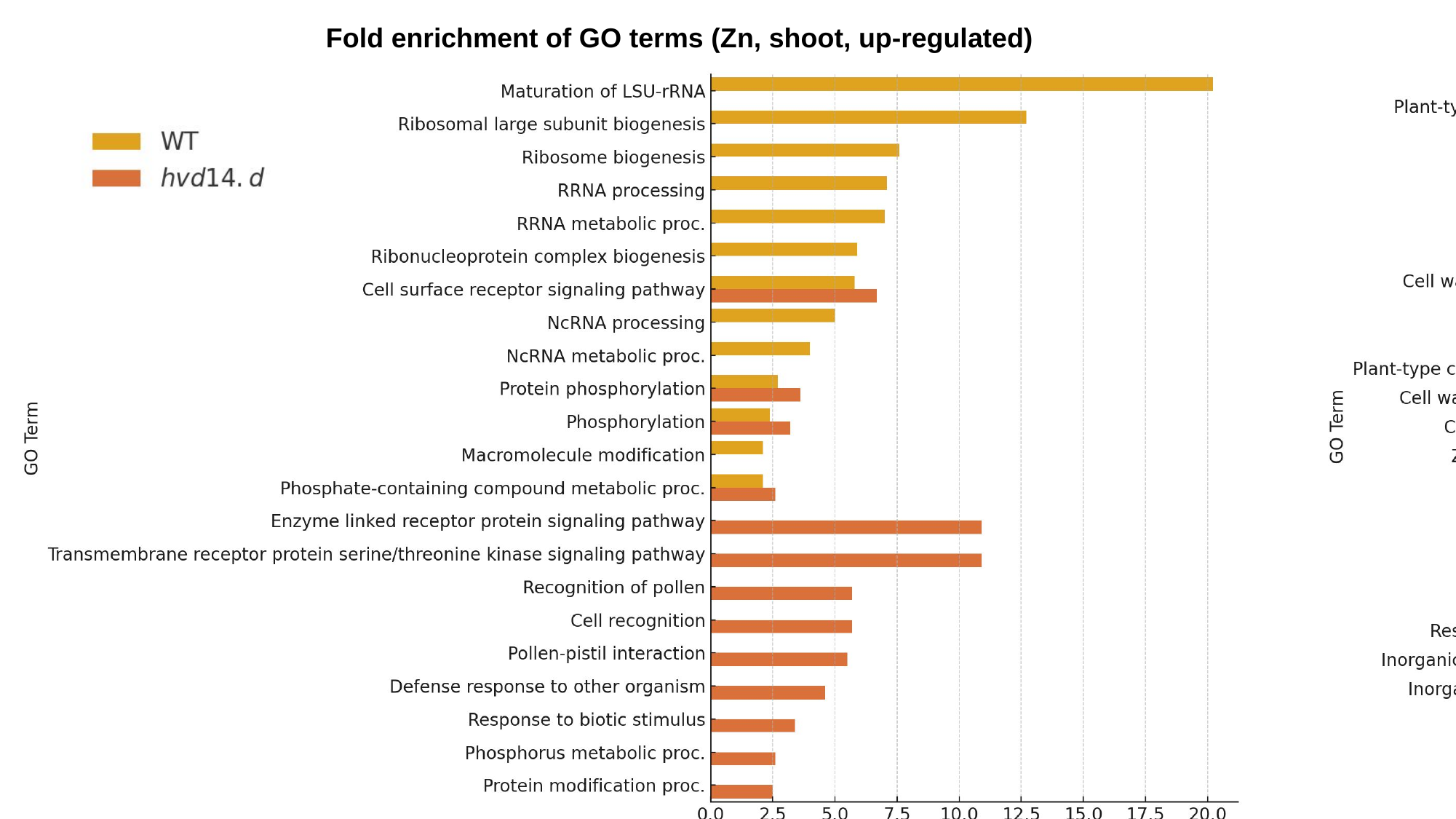

Fig. S4
Fold enrichment of GO terms (Zn, shoot, down-regulated)
Fold enrichment of GO terms (Zn, shoot, up-regulated)
#
Fold enrichment of GO terms (Zn, root, down-regulated)
Fold enrichment of GO terms (Zn, root, up-regulated)

## Slide 6
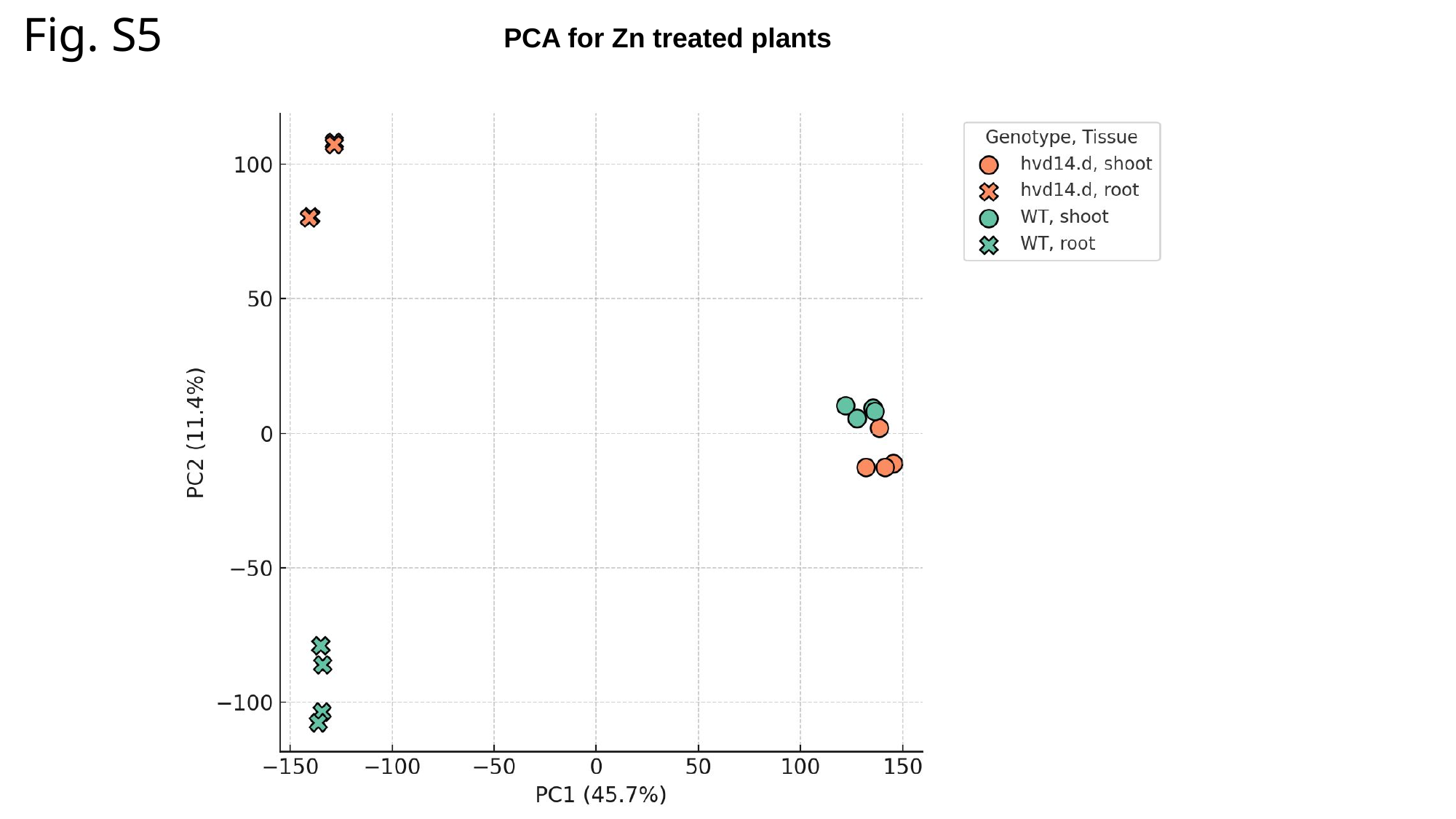

Fig. S5
PCA for Zn treated plants

## Slide 7
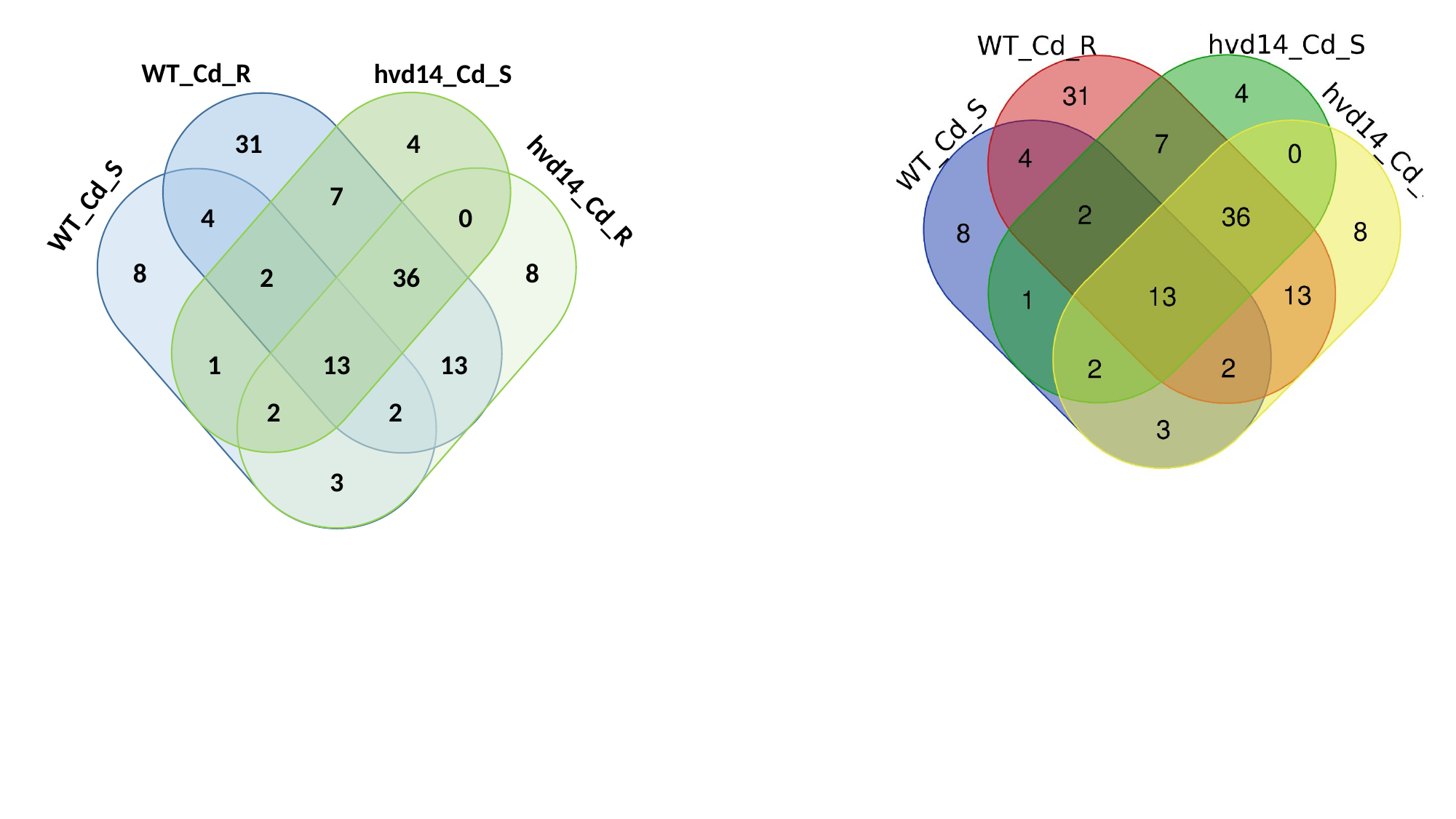

WT_Cd_R
hvd14_Cd_S
31
4
7
4
0
8
8
2
36
1
13
13
2
2
3
hvd14_Cd_R
WT_Cd_S

## Slide 8
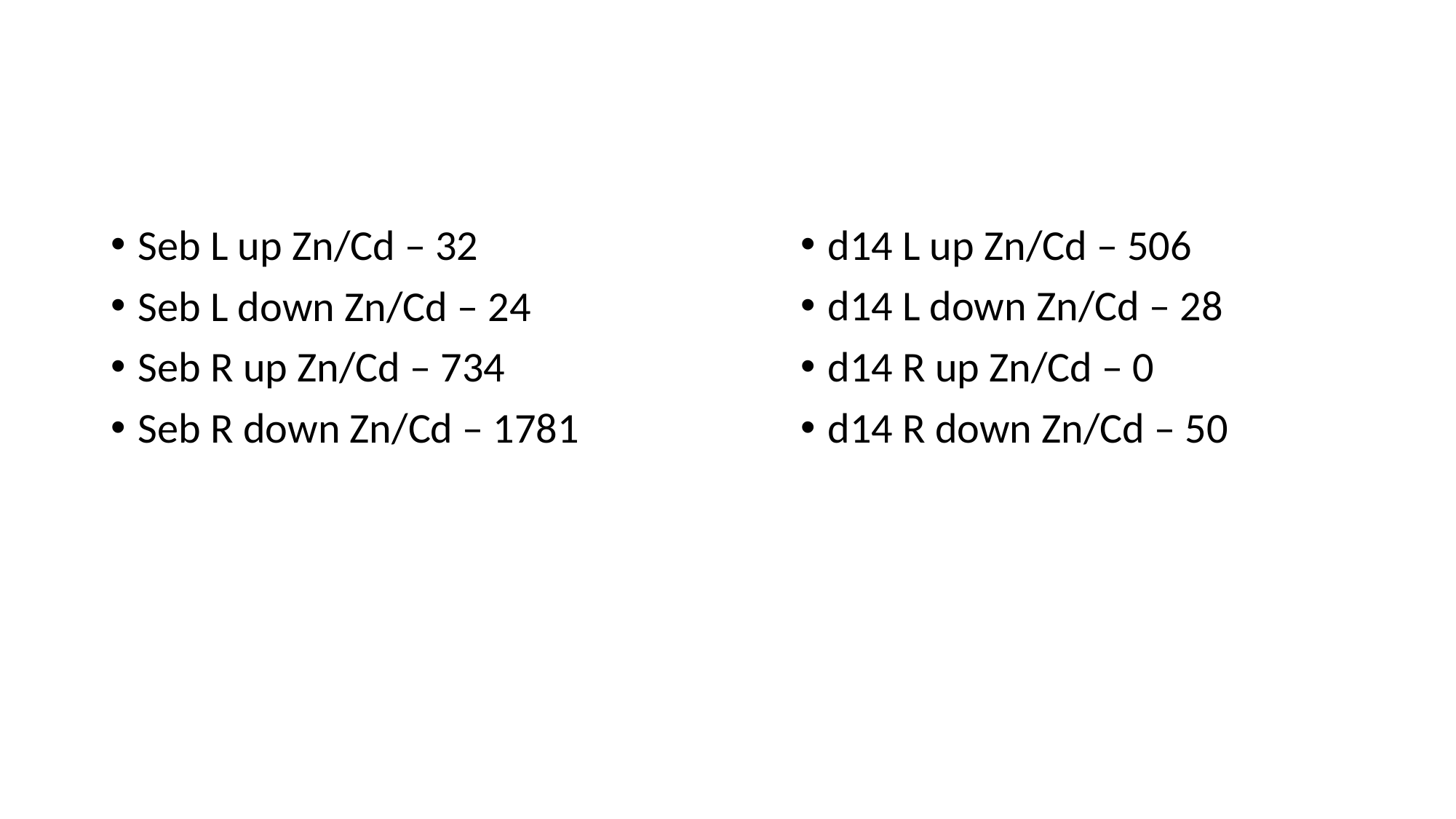

Seb L up Zn/Cd – 32
Seb L down Zn/Cd – 24
Seb R up Zn/Cd – 734
Seb R down Zn/Cd – 1781
d14 L up Zn/Cd – 506
d14 L down Zn/Cd – 28
d14 R up Zn/Cd – 0
d14 R down Zn/Cd – 50

## Slide 9
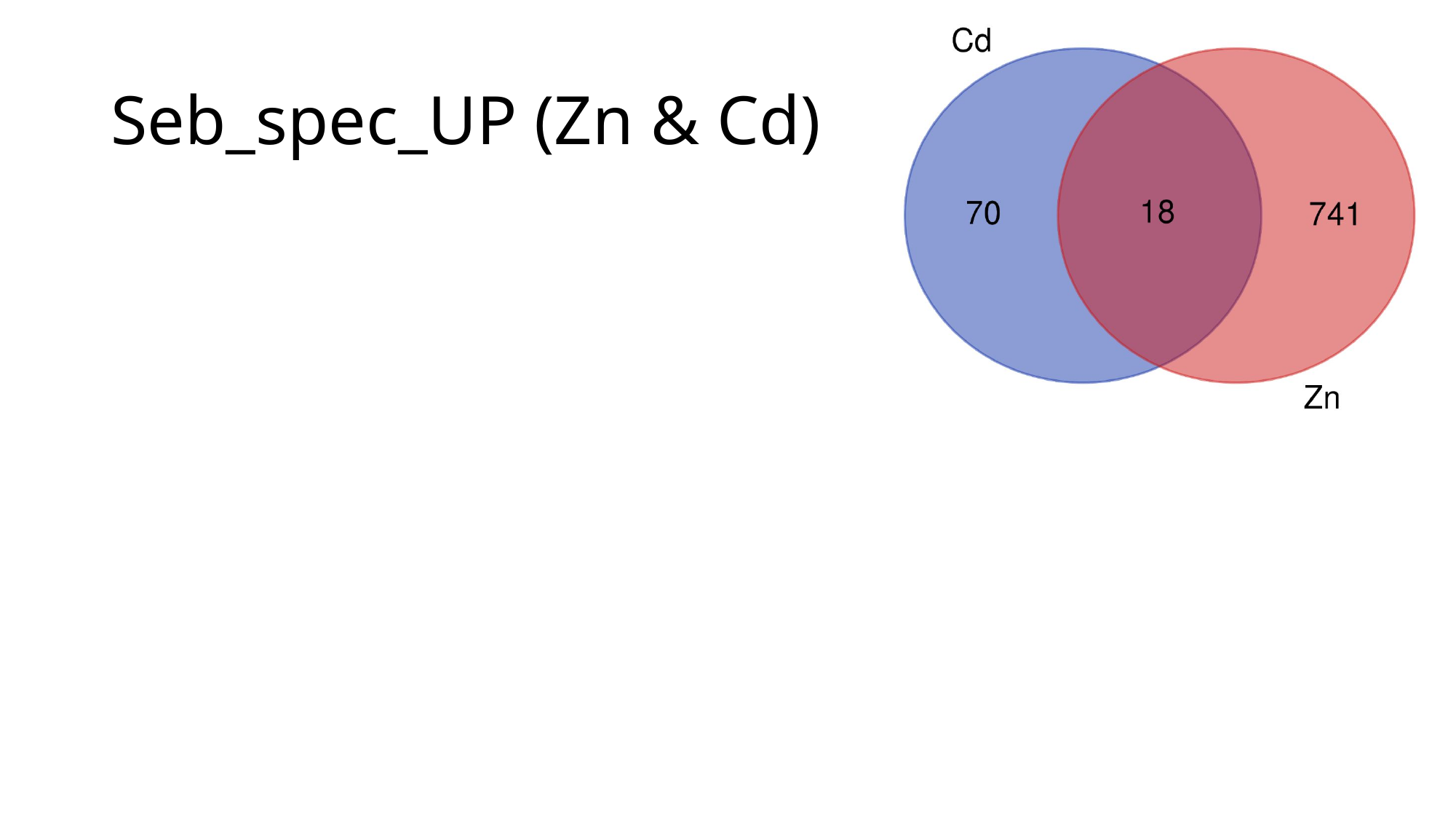

# Seb_spec_UP (Zn & Cd)

## Slide 10
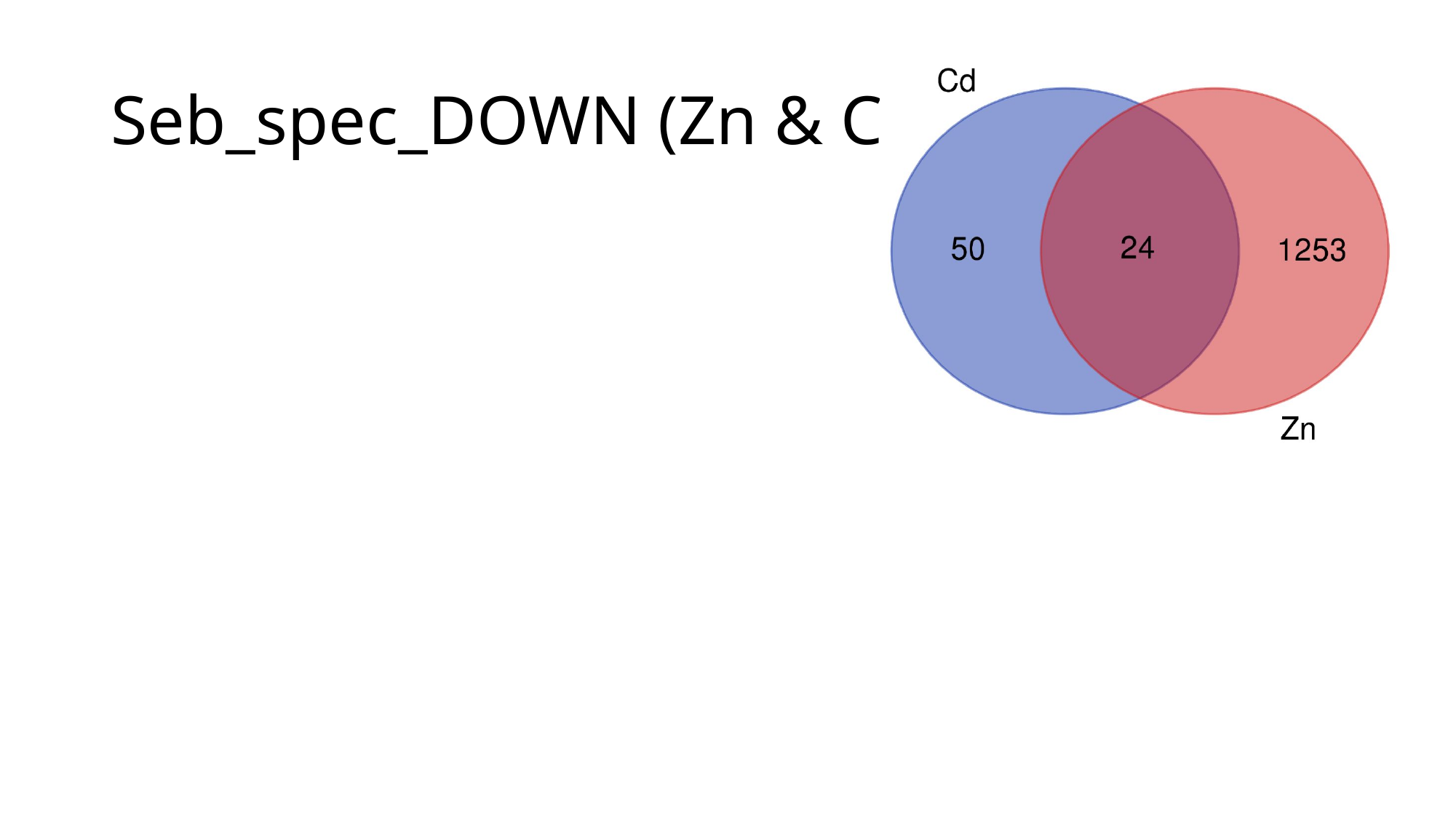

# Seb_spec_DOWN (Zn & Cd)

## Slide 11
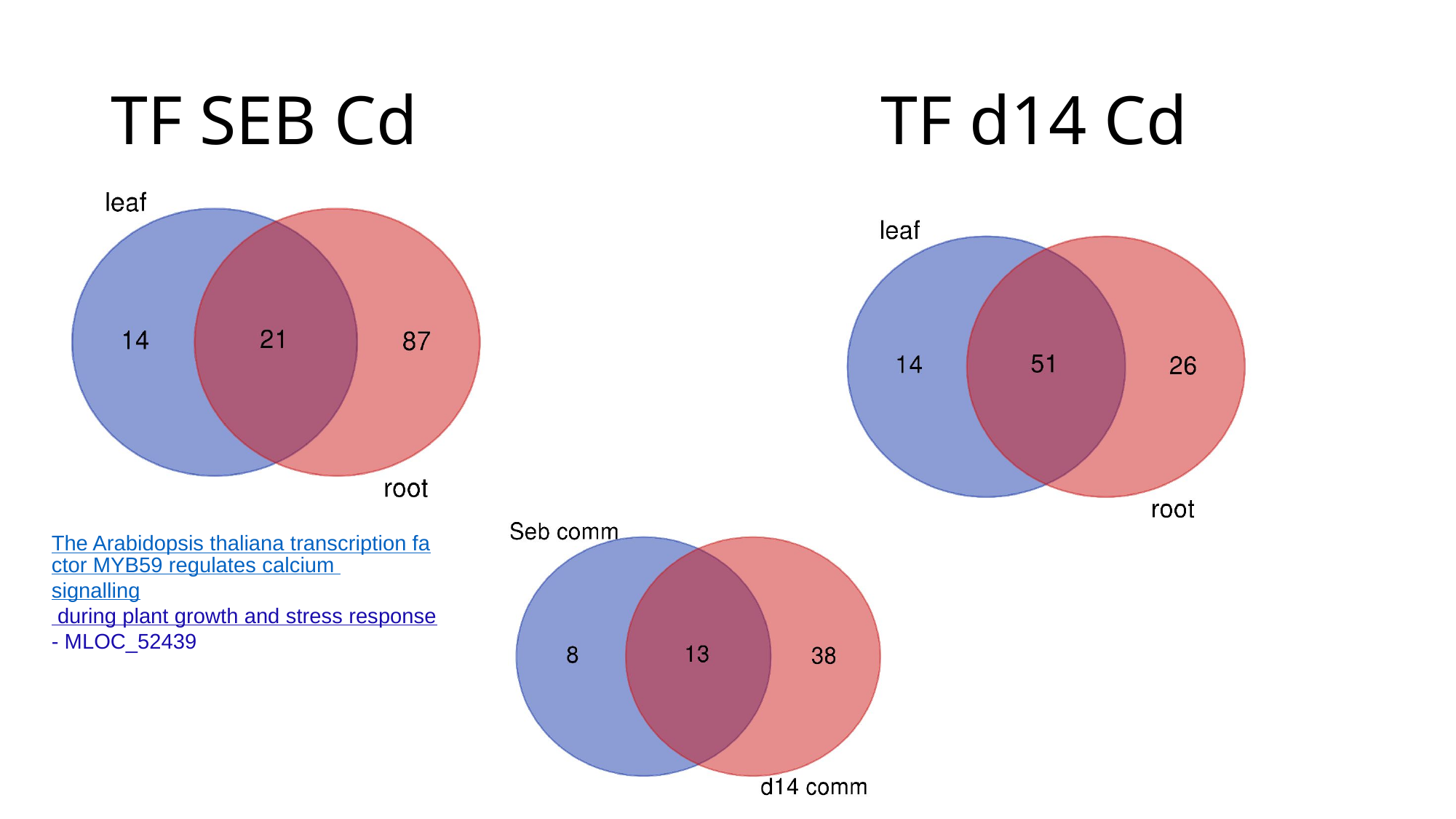

# TF SEB Cd
TF d14 Cd
The Arabidopsis thaliana transcription factor MYB59 regulates calcium signalling during plant growth and stress response - MLOC_52439
